# Oxidation-derived metabolites sustain the antioxidant network of quercetin

**DOI:** 10.1007/s10822-026-00878-3

**Published:** 2026-07-18

**Authors:** Yue Wang, Zhengwen Li, Ming Zhang, Berta Cillero-Pastor

**Affiliations:** 1 Department of Cell Biology-Inspired Tissue Engineering, MERLN Institute for Technology-Inspired Regenerative Medicine, Maastricht University, 6200 MD Maastricht, The Netherlands; 2 Nutrition and Translational Research in Metabolism (NUTRIM), Maastricht University, 6200 MD Maastricht, The Netherlands; 3 School of Pharmacy, Chengdu University, Chengdu, 610106 China; 4 Hainan University-HSF/LWL Collaborative Innovation Laboratory, College of Food Sciences & Engineering, Hainan University, Haikou, 570228 China; 5 Maastricht MultiModal Molecular Imaging Institute (M4I), Maastricht University, 6200 MD Maastricht, The Netherlands

**Keywords:** Quercetin, Unique antioxidant network, Density functional theory, Network pharmacology, Molecular docking

## Abstract

Quercetin is a widely consumed dietary flavonoid and nutraceutical with diverse biological activities, yet the mechanisms underlying its potent antioxidant effects remain incompletely understood. This study aims to examine the hypothesis that quercetin’s antioxidant efficacy is associated with a unique coordinating network in which radical-derived oxidation metabolites retain reducing capacity, supporting successive scavenging reactions and potentially contributing to selected regulatory pathways.

To investigate this hypothesis, oxidation metabolites were first generated using 2,2-diphenyl-1-picrylhydrazyl as a readily monitored and well-controlled radical oxidant. The reaction mixture was separated using an Agilent 1290 Infinity ultrahigh-performance liquid chromatography system with a water–acetonitrile gradient. Metabolites of interest were then characterized using a quadrupole time-of-flight tandem mass spectrometer in negative-ion mode and annotated based on [M−H]⁻ and fragmentation patterns. Electronic-structure and thermodynamic calculations based on density functional theory (DFT) were performed to evaluate frontier molecular orbitals and the mechanistic feasibility of the common antioxidant pathways. These results indicate that these metabolites can retain, and in some cases exceed the radical-scavenging capacity of the parent molecule. Network pharmacology analysis and molecular docking further suggest that these metabolites potentially engage a substantial portion of quercetin’s core regulatory targets and may contribute to its broader biological effects. Notably, among these metabolites, 2-benzoyl-2-hydroxy-3(2H)-benzofuranones emerged as promise.

Explanatorily, time-dependent density functional theory (TD-DFT) analysis further suggested that the formation of successively reactive metabolites may be facilitated by the characteristic excited-state intramolecular proton-transfer mechanism of flavonols. These findings warrant further investigation of oxidation metabolites within quercetin’s antioxidant network.

**Graphical abstract:**

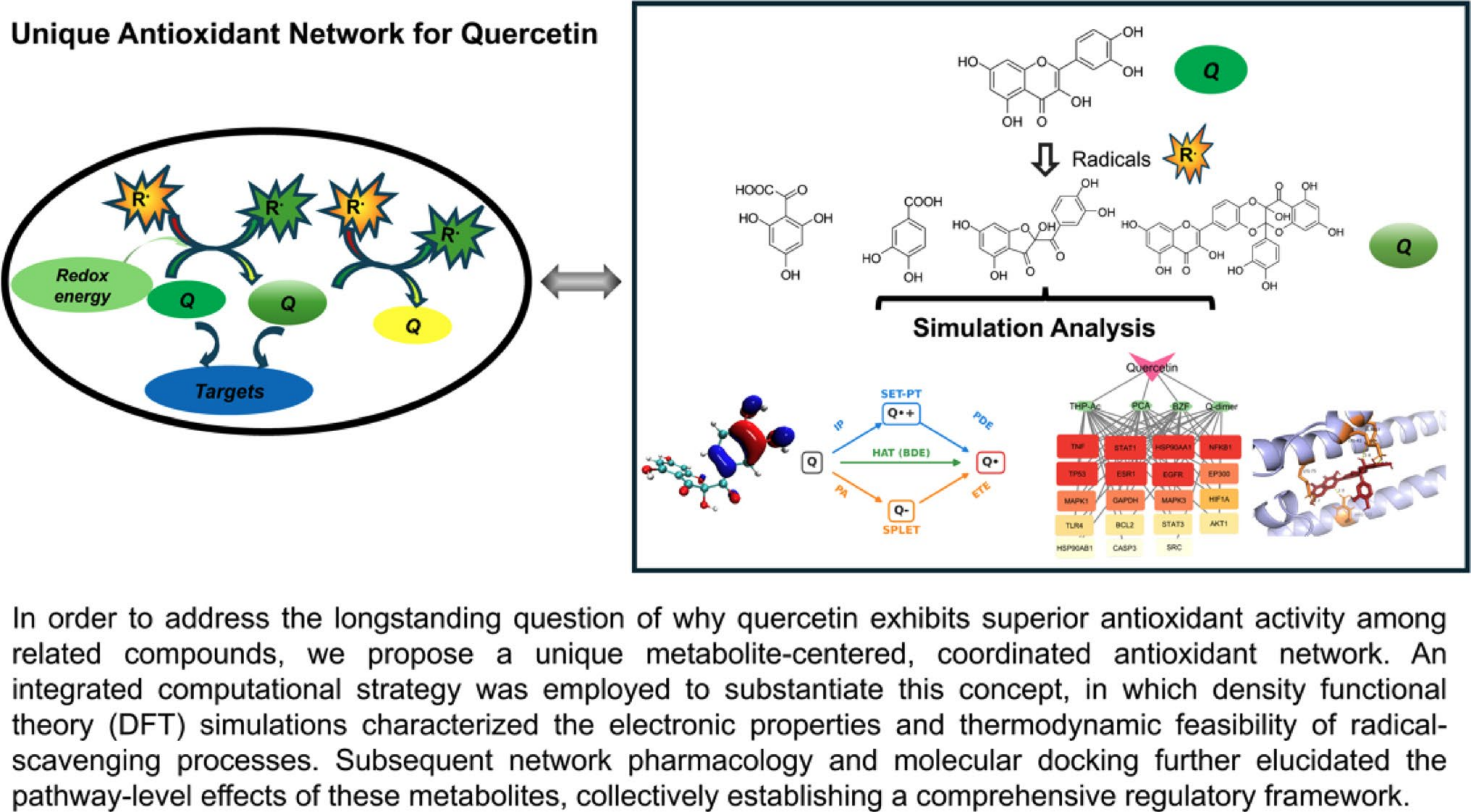

**Supplementary Information:**

The online version contains supplementary material available at 10.1007/s10822-026-00878-3.

## Introduction

Redox imbalance is increasingly recognized as a fundamental driver in the initiation and progression of numerous pathological conditions [1]. This recognition has spurred growing interest in natural molecules capable of modulating redox processes, particularly dietary polyphenols [2]. Among them, quercetin (Q) stands out for its potent and extensive pharmacological activities, exceeding 23 reported functions, including antioxidant, anti-inflammatory, cardioprotective, neuroprotective, and anticancer effects [3]. These properties, along with its natural abundance, have facilitated its widespread use as a nutritional supplement [4].

Paradoxically, despite extensive experimental evidence supporting the potent biological activities of Q, its true physiological relevance remains a matter of debate. Despite substantial dietary consumption, the circulating level of Q typically remains within the nanomolar range. Plasma concentrations in the micromolar range have generally been observed only after supplementation with pharmacologically high doses, such as 1000 mg of Q per day [5]. This limited systemic exposure is mainly attributed to its poor aqueous solubility, restricted intestinal uptake, rapid presystemic metabolism, and active efflux transport [6]. Accordingly, despite extensive clinical investigation, the ClinicalTrials.gov database (http://www.clinicaltrials.gov) records 121 registered studies involving Q thus far, covering a wide spectrum of biological activities and diverse therapeutic delivery strategies, including liposomes complexes, cyclodextrin inclusion complexes, and self-nanoemulsion drug delivery systems (SNEDDS) [7]. Nevertheless, it has not been approved to date for any therapeutic indication [8].

Recent studies have increasingly investigated Q analogues and derivatives as distinct classes of compounds that may offer improved solubility, absorption, metabolic stability, or bioactivity [9]. Structurally, Q is a 3,5,7,3′,4′-pentahydroxyflavonol aglycone characterized by the C6–C3–C6 flavonoid framework, comprising two aromatic rings (A and B) linked through an oxygen-containing heterocyclic C ring. Closely related flavonol analogues retain this core scaffold but differ in their hydroxylation patterns. Although these structural differences are relatively small, they can substantially affect antioxidant behavior by altering electron distribution, intramolecular hydrogen bonding, radical stabilization, and susceptibility to oxidation [10]. For example, kaempferol, which only lacks Q’s 3′-hydroxyl group, showed much weaker DPPH ((2,2-diphenyl-1-picrylhydrazyl)) radical-scavenging activity, with an IC_50_ of 5.32 μg/mL versus 1.84 μg/mL for Q [11]. The most extensively studied Q derivatives are formed through modification of its hydroxyl groups, while glucuronidated, sulfated, and methylated conjugates are also generated through phase II metabolism after absorption [12]. Such modifications may improve solubility, metabolic stability, or systemic durability; however, they typically retain only a portion of the aglycone's antioxidant activity. Lesjak et al. reported DPPH IC_50_ values below 1.33 μg/mL for Q, approximately 3.3–3.7 μg/mL for isorhamnetin and tamarixetin, 58 μg/mL for isorhamnetin-3-O-glucoside, and above 111 μg/mL for quercetin-3,4′-di-O-glucoside [13]. A recent review summarized 11 naturally occurring Q derivatives investigated for their antioxidant and anti-inflammatory properties, including isoquercitrin, rutin, quercitrin, hyperoside, isorhamnetin, and tamarixetin [14]. Nevertheless, no individual analogue, derivative, or phase II metabolite has demonstrated consistent superiority over Q aglycone across pharmacokinetic performance and bioactivity. A detailed understanding of the intrinsic antioxidant and oxidation mechanisms of Q aglycone therefore remains essential for interpreting the biological behavior of its structurally related compounds.

It is well established that Q confers antioxidant protection through two complementary modes of action: a direct mechanism, in which it neutralizes reactive species; and an indirect mechanism, in which it regulates diverse cytoprotective pathways through target engagement and network-level interactions [15]. The catechol moiety in the B ring, the C2 = C3 double bond conjugated with the 4-oxo group, and the 3- and 5-hydroxyl groups promote electron delocalization and stabilization of the resulting phenoxyl radicals [16, 17]. Accordingly, Q can neutralize reactive species through hydrogen-atom transfer, sequential proton-loss electron transfer, and single-electron transfer followed by proton transfer, with the preferred mechanism depending on the solvent, pH, radical identity, and ionization state [18–20]. These reactions generate semiquinone radicals, quinone or quinone-methide intermediates, and subsequently a range of oxidation products [21]. In parallel, Q may enhance cellular antioxidant capacity by modulating redox-sensitive signaling pathways. For example, activation of the Kelch-like ECH-associated protein 1–nuclear factor erythroid 2-related factor 2–antioxidant response element (Keap1–Nrf2–ARE) pathway promotes the transcription of cytoprotective enzymes, including heme oxygenase-1, NAD(P)H oxidoreductase 1, glutamate–cysteine ligase, and enzymes involved in glutathione metabolism [22, 23]. Q has also been reported to modulate nuclear factor kappa B (NF-κB), mitogen-activated protein kinase (MAPK), phosphoinositide 3-kinase–protein kinase B (PI3K–Akt), and adenosine monophosphate-activated protein kinase (AMPK) signaling, thereby influencing inflammatory responses, mitochondrial homeostasis, apoptosis, and cellular adaptation to oxidative stress [24–26].

Although direct chemical and indirect regulatory mechanisms are often regarded as parallel and largely independent, this distinction may be overly simplified. This possibility is highlighted by Q’s behavior under moderate oxidation [21, 27]. Instead of showing a clear loss of antioxidant activity after oxidation, Q-derived oxidation mixtures have been reported to display greater cell-protective activity than the parent compound [28]. These observations suggest that direct and indirect modes of action may be partially interconnected rather than entirely independent. We therefore hypothesize that radical-mediated oxidation of Q may facilitate the formation of oxidative metabolites that contribute to its overall antioxidant profile. These metabolites may further support subsequent radical-scavenging reactions and potentially contribute to selected regulatory pathways related to antioxidant responses. Together with the parent compound, these processes may form an interconnected antioxidant framework that helps explain the pronounced antioxidant activity of Q.

To gain a comprehensive understanding of this synergistic framework, experimental approaches alone are limited in both efficiency and scope, owing to the involvement of various compounds, molecular targets, and pathways. Accordingly, the present study employs computational simulation strategies, which have emerged as powerful and versatile tools for elucidating the complex biological behavior of flavonoids, including Q [29]. Specifically, density functional theory (DFT) has been extensively applied to provide high-resolution mechanistic insights into radical scavenging processes [19, 30]. Complementarily, bioinformatics-driven computational data mining enables the systematic characterization of target engagement and offers an integrated view of the biological regulatory networks [31].

The basis of this hypothesis is that radical-mediated oxidation of Q could generate early-stage oxidative metabolites that may retain the capacity to participate in subsequent redox reactions. Accordingly, this study adopts a stepwise strategy to identify these metabolites and then evaluate their antioxidant potential. The DPPH was selected as a stable and readily monitored radical oxidant for generating early-stage oxidation products. Upon accepting electrons or hydrogen atoms from Q, the violet DPPH radical is converted to its reduced form, resulting in a measurable decrease in absorbance. In addition, the bulky phenyl and picryl groups surrounding the nitrogen-centered radical sterically restrict access to the reactive site, thereby moderating molecular encounters and slowing the reaction kinetics [32]. In contrast to conventional antioxidant assays that frequently employ an excess of DPPH [16, 33], an equimolar DPPH-to-Q ratio was used in this study to induce detectable oxidation while limiting extensive oxidation. Subsequently, DFT calculations were conducted to evaluate the radical-scavenging potential of presentative metabolites at the molecular level, whereas network pharmacology analysis and molecular docking were performed to characterize target engagement, associated biological networks, and binding affinities at the systems level. Finally, the molecular basis underlying this distinct network was discussed.

## Materials and methods

### Chemicals

Q (≥ 95%) and 2,2-diphenyl-1-picrylhydrazyl (DPPH·) was purchased from Sigma − Aldrich (St. Louis, MO). Ethanol (EtOH) was analytical grade. Formic acid and acetonitrile were ultrahigh performance liquid chromatography (UPLC) grades.

### Preparation of oxidative metabolites

Q and DPPH· reacted at a 1:1 molar ratio in ethanol. Q was dissolved in EtOH (with 5% DMSO) to prepare a 200 μM stock solution. DPPH· was freshly prepared in EtOH at 200 μM and protected from light. 500 μL of the two stock solutions were mixed to obtain a final reaction mixture. The solution was vortexed and incubated for 10 min at room temperature in the dark, then 10 μL of 1 M HCl was added to terminate the reaction.

### Identification of major oxidative metabolites

The product mixture was separated using an Agilent 1290 infinity ultrahigh-performance liquid chromatography (UPLC) system (Agilent Technologies, Santa Clara (CA), USA). The UPLC was equipped with a quaternary pump with an integrated vacuum degasser (G4204A), an autosampler (G4226A) with a thermostat (G1330B), a diode array detector (G4212A), and a thermostatic column compartment (G1316C). The column used was a Waters Xbridge BEH-C18 4.6 × 100 mm (3.5 µm particle size). The mobile phase consisted of water (solvent A), acetonitrile (solvent B), and 2% (v/v) formic acid in water (solvent C), with a flow rate of 0.8 mL/min. The gradient elution profile was as follows: 0–1 min, 5% B; 1–4 min, 5–7.5% B; 4–11 min, 7.5–70% B; 11–12.5 min, 70–95% B. UV detection was performed at 254, 294, and 370 nm. The wavelengths of 254 and 370 nm were selected to monitor the characteristic flavonoid absorption regions, whereas 294 nm was included because preliminary spectral examination revealed an increase in absorbance at this wavelength during quercetin oxidation.

The analytes were detected using an Agilent 6550 iFunnel Quadrupole Time-of-Flight Mass Spectrometer (Q-TOF LC–MS/MS) equipped with Dual Agilent Jet Stream electrospray ionization source (Dual AJS ESI) set to negative ionization mode. The capillary voltage was set to -3500 V. The nebulizer pressure was 35 psi. Mass spectra were acquired over an m/z range of 50–750 in all-ions MS/MS mode using collision energies of 0, 10, and 20 V. The 0 V channel provided precursor-ion accurate-mass information, whereas the 10 and 20 V channels generated fragment-ion information. Data was processed using Agilent MassHunter Qualitative Analysis. Molecular formulas were tentatively generated with a mass tolerance of ± 5 ppm, with the elemental composition restricted to C, H, and O within predefined atom-number limits. In negative-ion mode, [M − H]⁻ was considered the principal ion species. Compound assignments were based on accurate-mass and all-ions MS/MS fragmentation data, supported, where applicable, by mzCloud spectral-library (https://www.mzcloud.org/) matching, NMR characterization, and comparison with previously reported mass-spectrometric data. Proton nuclear magnetic resonance (^1^H NMR) and carbon-13 nuclear magnetic resonance (^13^C NMR) spectroscopy in dimethyl sulfoxide-d6 (DMSO-d_6_), recorded at 600 and 125 MHz, respectively.

### Computational simulation

#### Systematic conformation searching

Initial geometry construction and comprehensive conformational search was performed in Spartan’14 (v1.1.4) using the Merck Molecular Force Field (MMFF). The rotatable single bonds were defined as torsional variables. Subsequently, systematic conformer exploration was performed using a sixfold rotational sampling strategy with 60° increments. The dominant conformer with the lowest energy were selected for subsequent optimization.

#### Geometry optimizations and single-point energy calculation

The high-level geometry optimizations were subsequently carried out in Gaussian 16. M062X functional was employed due to it is well suited for small-molecule activity analysis [34]. The 6–311 ++ G(d,p) basis set was selected to provide reliable prediction of the thermodynamic parameters and electronic properties associated with antioxidant activity. The polarization functions ("d,p"), which introduce higher-angular-momentum functions beyond the valence shell, improve the flexibility of the electron density description by allowing the orbitals to better respond to bonding interactions and changes in the molecular environment [35]. In addition, the diffuse functions (“ ++ ”) are particularly important in the present study for properly describing radical and anionic species, in which the unpaired or excess electrons are more spatially extended and distributed farther from the atomic nuclei [36]. Single-point energy calculations were performed at the aug-cc-pVDZ level to obtain more reliable relative energies. This computational combination has been demonstrated to perform well in predicting the reaction enthalpies associated with hydrogen atom transfer (HAT), single-electron transfer followed by proton transfer (SET-PT), and sequential proton loss electron transfer (SPLET) mechanisms in phenolic antioxidant compounds [37].

#### Electronic properties analysis

The electronic distribution of the molecules was represented by the frontier molecular orbitals (FMOs), including the highest occupied molecular orbital (HOMO) and the lowest unoccupied molecular orbital (LUMO). The HOMO represents the electron-rich regions of the molecule and reflects the potential to donate electrons, whereas the LUMO represents the electron-deficient regions and indicates capability of accepting electrons [38]. Visualization was carried out using Multiwfn 3.8 [39] in combination with VMD 1.9.4. The frontier molecular orbital energy gap (${E}_{\mathrm{g}\mathrm{a}\mathrm{p}}$) were further calculated as Eq. (1), to comprehensively evaluate the and chemical reactivity.

1 $${E}_{gap}={E}_{LUMO}-{E}_{HOMO}$$

#### Thermodynamic analysis

The enthalpy changes associated with the classical antioxidant mechanisms, including HAT, sequential proton SPLET, and SET-PT, were evaluated to assess the thermodynamic feasibility of different molecules [40]. The enthalpies (H) of compounds were calculated using Shermo 2.6.1 [41], by combining the above single-point electronic energies with thermal corrections derived from the frequency calculations. Grimme’s quasi-rigid rotor harmonic oscillator (quasi-RRHO) model was applied to treat low-frequency vibrational modes, and zero-point energy corrections were included using a scaling factor of 0.9877 [42] [43]. The solution-phase enthalpies of the hydrogen atom, proton and electron were taken as − 98.8, 1052.7 kJ/mol and −77.5 kJ/mol, respectively [44].

For the HAT mechanism, the bond dissociation enthalpy (BDE) was calculated according to Eq. (2):

2 $$BDE=H(Ar{O}^{\cdot })+H({H}^{\cdot })-H(ArOH)$$

For the SPLET mechanism, the proton affinity (PA) as Eq. (3):

First deprotonation:

3 $$P\mathrm{A}=H(Ar{O}^{-})+H({H}^{+})-H\left(ArOH\right)$$

For the SET-PT mechanism, the ionization potential (IP) as Eq. (4):

4 $$IP=H(ArO{H}^{\cdot +})+H({e}^{-})-H(ArOH)$$

where $H(ArOH)$, $H(Ar{O}^{\cdot })$, $H(ArO{H}^{\cdot +})$, and $H(Ar{O}^{-})$ represent the enthalpies of the neutral molecule, radical species, radical cation, and anionic species, respectively.

#### Time-dependent density functional theory (TD-DFT) and natural transition orbital (NTO) analysis

TD-DFT calculations were performed in Gaussian 16 using the same M06-2X/6–311 +  + G(d,p) level of theory and SMD water solvation model employed for geometry optimization and frontier molecular orbital analysis. Vertical excitation energies were calculated from the optimized ground-state geometries, followed by geometry optimization of the first singlet excited state (S1). NTO analysis was performed using Multiwfn based on the TD-DFT transition density matrices to characterize the dominant hole and electron distributions before and after proton transfer. LUMOs were further evaluated for the resulting ground-state structures using the same computational level.

### Network pharmacology analysis

#### Target retrieval

The GeneCards database (https://www.genecards.org) was utilized to identify targets of oxidative stress. Known target information for Q was compiled from both the GeneCards database and CHEMBL (https://www.ebi.ac.uk/chembl/). The structures of the main metabolites were entered into several target prediction databases, including Passtargets (https://www.way2drug.com/passtargets/), ppb2 (https://ppb2.gdb.tools/), SEA (https://sea.bkslab.org/), Swiss Target Prediction (http://www.swisstargetprediction.ch/), and SuperPred (https://prediction.charite.de/), to generate a potential target ensemble with a prediction probability threshold of > 50%. All targets were then standardized using the UniProt database (http://www.uniprot.org/). Intersection patterns among the target sets were visualized in R version 4.4.3 with the *UpSetR* package.

#### Network relationship construction

The Protein–Protein Interaction (PPI) network was constructed through the STRING database (https://string-db.org/). Interactions meeting the high-confidence threshold (confidence score ≥ 0.700) were subsequently imported into Cytoscape 3.10.2 for network visualization and analysis. Core targets were identified through three iterative screening cycles using six topological algorithms implemented in the CytoHCA plug-in: betweenness centrality (BC), closeness centrality (CC), degree centrality (DC), eigenvector centrality (EC), local average connectivity (LAC), and network centrality (NC). For each algorithm, the median score was calculated, and nodes with values exceeding the median were retained. Subsequently, the top 10% of nodes were selected as top targets based on degree values. The targets' overlaps were visualized in R 4.4.3 with *VennDiagram* package.

### Molecular docking

Protein crystal structures were obtained from the RCSB Protein Data Bank (PDB). Experimentally determined “Homo sapiens” protein structures with crystallographic resolution better than 2.5 Å and co-crystallized small-molecule ligands were selected for docking. Protein structures were pre-processed in PyMOL 3.0 by recording the ligand-center coordinates, removing crystallographic waters, and deleting non-essential heteroatoms. Receptors and ligands were prepared using AutoDockTools 1.5.7 by adding hydrogen, assigning Gasteiger charges, and defining AD4 atom types.

The docking protocol was validated by redocking each co-crystallized ligand into its native binding pocket using AutoDock Vina v1.2.x. Docking grids were centered on the recorded native ligand coordinates and adjusted to cover the binding pocket. Models with an RMSD below 2.0 Å between crystallographic and redocked poses were considered validated and used for subsequent docking. The ligand-center coordinates, docking box dimensions, and redocking RMSD values for all validated protein targets are provided in Supplementary Table S1.

Geometry-optimized Q and oxidation metabolite structures were docked into the validated targets using the same protocol. For the quercetin dimer, any grid dimension smaller than 25 Å was increased to accommodate its larger molecular size. Protein–ligand interactions were analyzed, and 2D interaction diagrams were generated using Discovery Studio Visualizer 2.0.

The binding affinity (ΔG, predicted docking binding energy) and ligand efficiency were evaluated to quantitatively characterize binding potential. Ligand efficiency was evaluated to partially normalize docking affinity scores with respect to molecular size, according to the following equation:

$$LE=\frac{-\Delta G}{{N}_{heavy}}$$

where ΔG represents the predicted docking binding energy (kcal/mol) and ${N}_{heavy}$ corresponds to the number of non-hydrogen (heavy) atoms in the ligand.

### Functional annotation

Kyoto Encyclopedia of Genes and Genomes (KEGG) pathway analyses were performed using in R 4.4.3 with *org.Hs.eg.db*, *enrichplot*, and *ggplot2* packages, pathways with *p* < 0.05 were retained. Disease-association scores were retrieved from the GeneCards Analysis module (https://ga.genecards.org/) and visualized as petal plots in R 4.4.3 with *ggplot2* package.

## Results

### Preparation and identification of oxidative metabolites

**Fig. 1:**
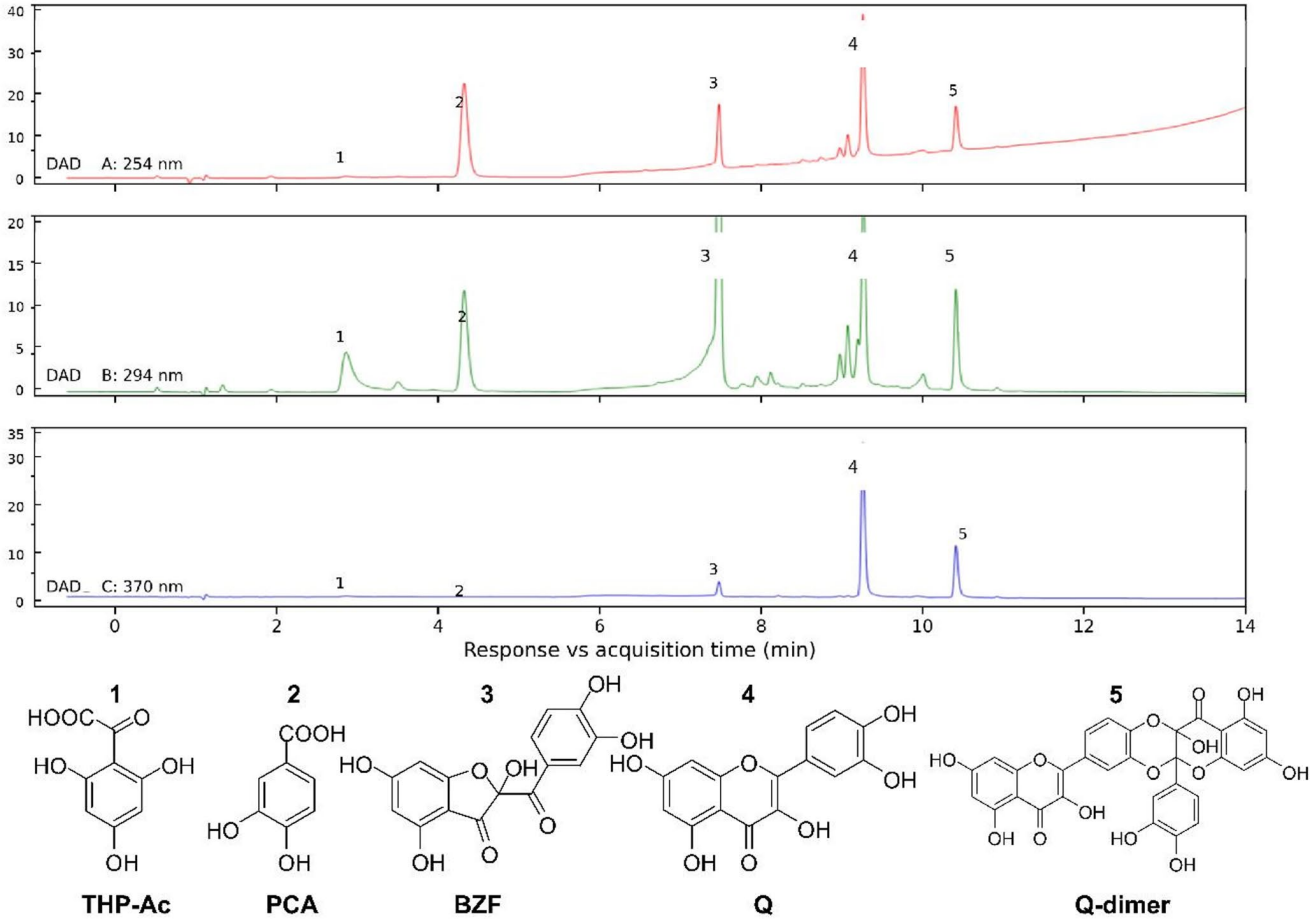
UPLC profiling of the quercetin (Q)–2,2-diphenyl-1-picrylhydrazyl (DPPH·) reaction mixture monitored at 254 nm (red), 295 nm (green), and 370 nm (blue), together with the corresponding structural characterization of the major peaks

To further investigate the potential roles of quercetin oxidation products within the antioxidant network, we first characterized their chemical structures. UPLC profiling of the Q–DPPH· reaction mixture at 254, 295, and 370 nm revealed five prominent chromatographic peaks (Fig. 1), their corresponding MS/MS spectra acquired at collision energies of 0, 10, and 20 V were shown in Supplementary Figure S1.

Peaks 1, 2, and 4 showed precursor ions at m/z 197, 153, and 301, with characteristic fragment ions at m/z 153/83, 109/108, and 179/151, respectively. Their tandem mass spectra closely matched the corresponding reference-library spectra and previously reported mass-spectrometric data, supporting their assignments as 2-oxo-2-(2,4,6-trihydroxyphenyl)acetic acid (THP-Ac), protocatechuic acid(PCA), and Q [45]. Peak 3 displayed a precursor ion at m/z 317 with characteristic fragment ions at m/z 190 and 163. Based on this fragmentation pattern and NMR characterization, it was assigned as 2-benzoyl-2-hydroxy-3(2H)-benzofuranone [27], with the corresponding NMR data provided in Supplementary Table S2. Peak 5 was assigned as a Q dimer based on its [M − H]⁻ ion at m/z 601 and characteristic fragment ions at m/z 301 and 299, consistent with previously reported Q oxidation data [46]. With the parent compound Q excluded, the remaining oxidative metabolites were collectively defined as Qox.

### Radical scavenging ability analysis

To obtain more reliable structural and reactivity predictions, we first evaluated the predominant protonation states of Q and Qox at pH 7.0–7.4 based on their reported or predicted pKa values. For Q, Luana et al. experimentally determined acidity constants of pKa_1_ = 8.29, pKa_2_ = 8.61, and pKa_3_ = 9.5 ± 0.1 using combined potentiometric and spectrophotometric measurements [47]. Based on the Henderson–Hasselbalch relationship, neutral Q remains the predominant species under near-physiological conditions (pH 7.0–7.4), while the population of monoanionic species increases from approximately 5% at pH 7.0 to ~ 11% at pH 7.4. Similar acid–base behavior is theoretically expected for BZF and Q-dimer because of their comparable polyphenolic hydroxyl environments. This assignment was further supported by ACD/Labs (https://www.acdlabs.com/products/percepta-platform/physchem-suite/pka/) site-specific pKa prediction. In contrast, PCA and THP-Ac predominantly exist as carboxylate monoanions under near-physiological conditions (> 95%), whereas additional phenolic hydroxyl deprotonation remains limited [48]. The experimentally determined and computationally predicted ionizable sites, pKa values, and predominant species at pH 7.0–7.4 are summarized in Supplementary Figure S2. Calculations were accordingly performed for dominant states of Q and Qox.

#### Electronic structure analysis

**Fig. 2:**
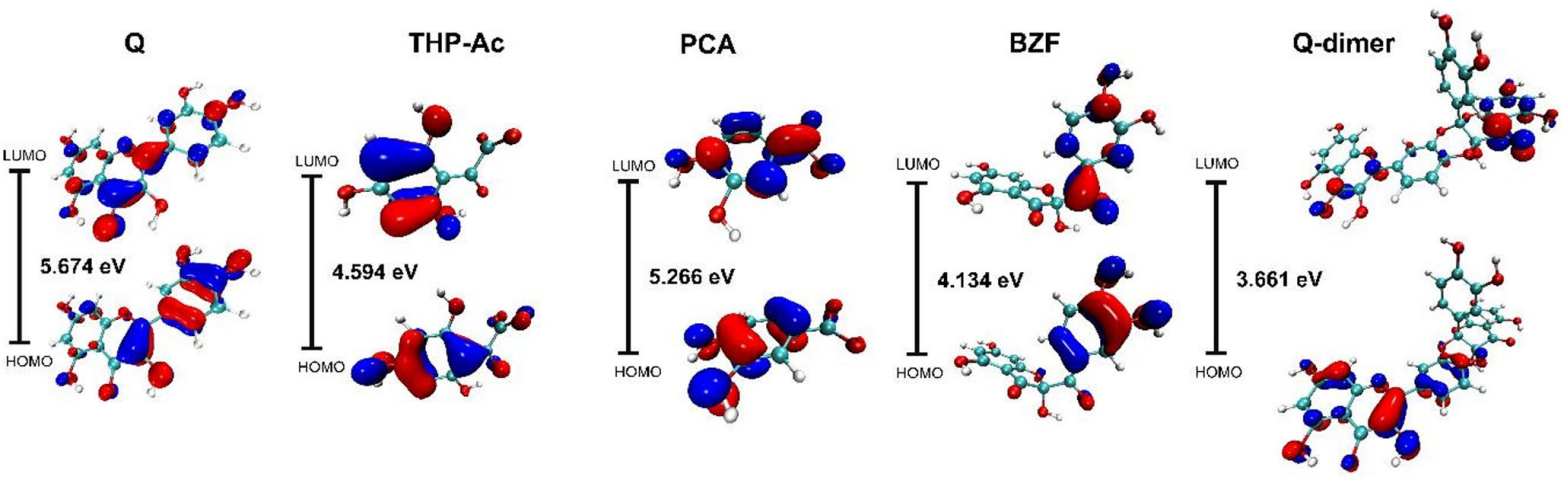
Frontier molecular orbitals and corresponding energy gaps of quercetin (Q) and oxidative metabolites ( THP-Ac, PCA, BZF, and the Q-dimer). Red represents α-spin orbitals and blue represents β-spin orbitals

To further assess the radical scavenging ability of Qox, two complementary computational approaches, electronic structure analysis and thermodynamic evaluation, were employed. The HOMO and LUMO distributions are shown in Fig. 2, to identify the regions involved in electron donation and acceptance, respectively. The corresponding HOMO–LUMO energy gaps were then calculated to evaluate electronic reactivity. The results showed that, all oxidation products exhibited smaller LUMO–HOMO gaps than Q (5.674 eV), suggesting enhanced electronic reactivity, with BZF (4.134 eV) and the Q-dimer (3.661 eV) showing the most pronounced decreases.

#### Thermodynamic analysis

**Fig. 3:**
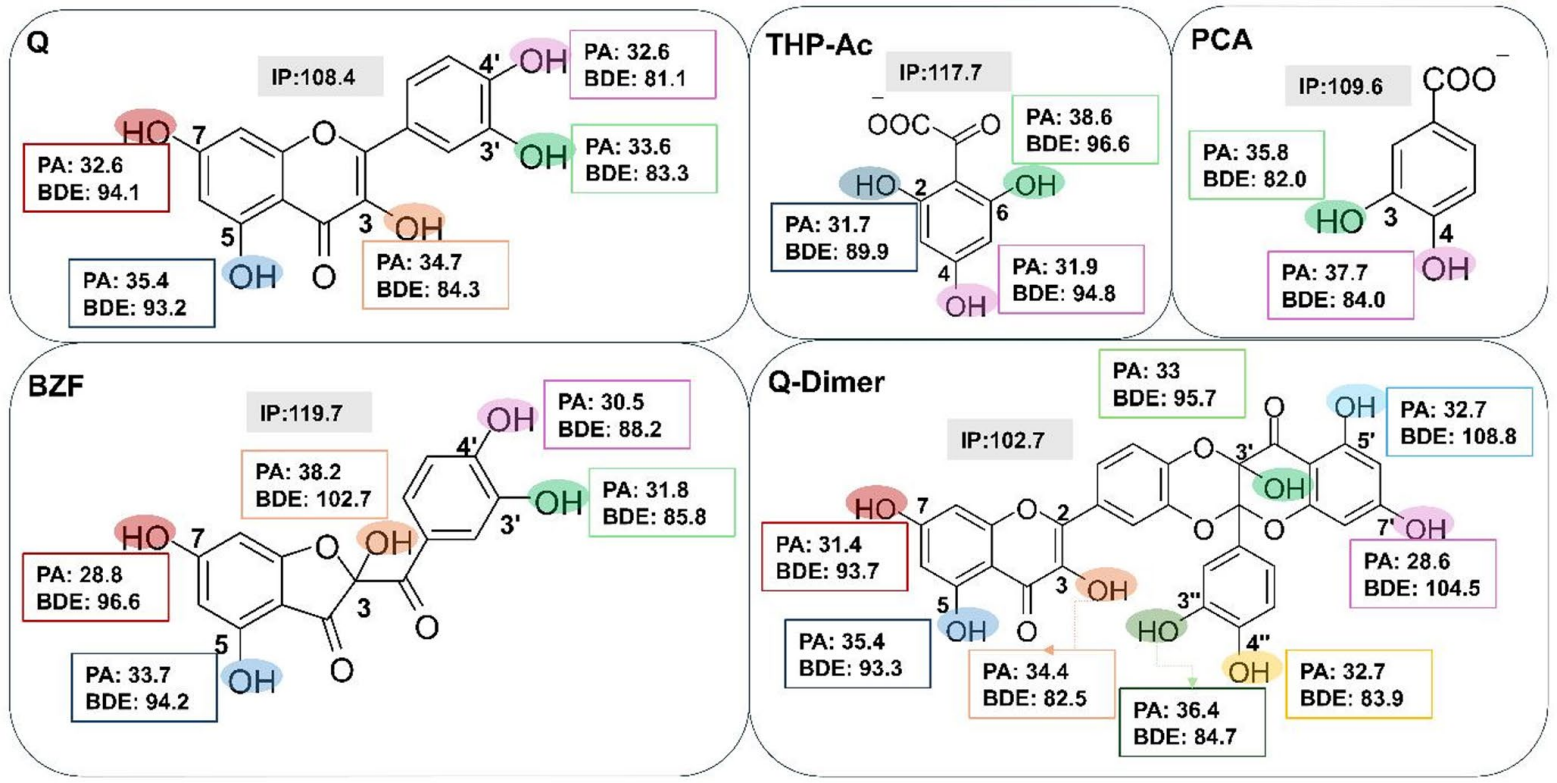
Enthalpies (kcal/mol) for quercetin (Q) and oxidative metabolites (THP-Ac, PCA, BZF, and the Q-dimer) in three representative radical scavenging mechanisms. BDE, bond dissociation enthalpy for the hydrogen atom transfer (HAT) mechanism; PA, proton affinity, for the sequential proton loss electron transfer (SPLET) mechanism; IP, ionization potential, for the single-electron transfer followed by proton transfer (SET-PT) mechanism

Thermodynamically, we compared the threshold enthalpy of three representative mechanisms to assess the radical-scavenging potential of Qox. As shown in Fig. 3, all compounds preferentially underwent proton dissociation in the initial stage, supporting SPLET as the dominant pathway in aqueous media. Although even for both THP-Ac and PCA were predicted to exist predominantly in their deprotonated forms in the water. Notably, the proton affinities (PA) of all phenolic hydroxyl groups in BZF were consistently lower than the corresponding values in Q, indicating greater intrinsic SPLET reactivity. THP-Ac also showed lower PA values at its 2-OH and 4-OH positions (31.7 and 31.9 kcal/mol) than Q’s most reactive 7/4’-OH site. In contrast, Q displays the lowest bond dissociation enthalpy (BDE) at its 4’-OH (81.1 kcal/mol), indicating its strong HAT-driven scavenging ability. For electron-transfer pathways, the relative ease follows: Q-dimer (102.7 kcal/mol) > Q (108.4 kcal/mol) > THP-Ac (109.6 kcal/mol) > PCA (117.7 kcal/mol) > BZF (119.7 kcal/mol). Collectively, these thermodynamic results indicated that although Q holds an advantage under HAT, Qox—especially BZF—displayed stronger intrinsic SPLET and ET-PT reactivity in aqueous environment. Taken together, the electronic-structure and thermodynamic analysis provided compelling evidence that Qox possessed substantial potential to participate in successive radical-scavenging processes. Importantly, BZF exhibits a markedly reduced frontier-orbital energy gap along with consistently lower PA values, underscoring its enhanced reactivity.

### Regulatory network involvement analysis of oxidative metabolites

#### Target engagement analysis

To further elucidate the involvement of Qox in the Q-driven oxidative stress (OS) regulatory network (Q-OS network), the associated target sets were first identified. Potential targets were retrieved from multiple databases (Table 1). A total of 13,632 related OS targets were initially compiled, and after merging data sources and removing duplicates, 1676 targets were retained for Q. Likewise, 923, 1504, 879, and 767 candidate targets were identified for THP-Ac, PCA, BZF, and the Q-dimer, respectively.

**Table 1:** Overview of potential targets retrieved from multiple databases

Database	OS	Q	THP-Ac	PCA	BZF	Q-dimer
GeneCards	13,632	859	–	–	–	–
CHEMBL	–	1035	–	–	–	–
Passtargets	–	–	694	1037	515	299
ppb2	–	–	184	129	192	206
SEA	–	–	20	442	65	116
Swiss target	–	–	17	51	15	12
SuperPred	–	–	194	233	280	345
Total	13,632	1676	923	1504	879	767

**Fig. 4:**
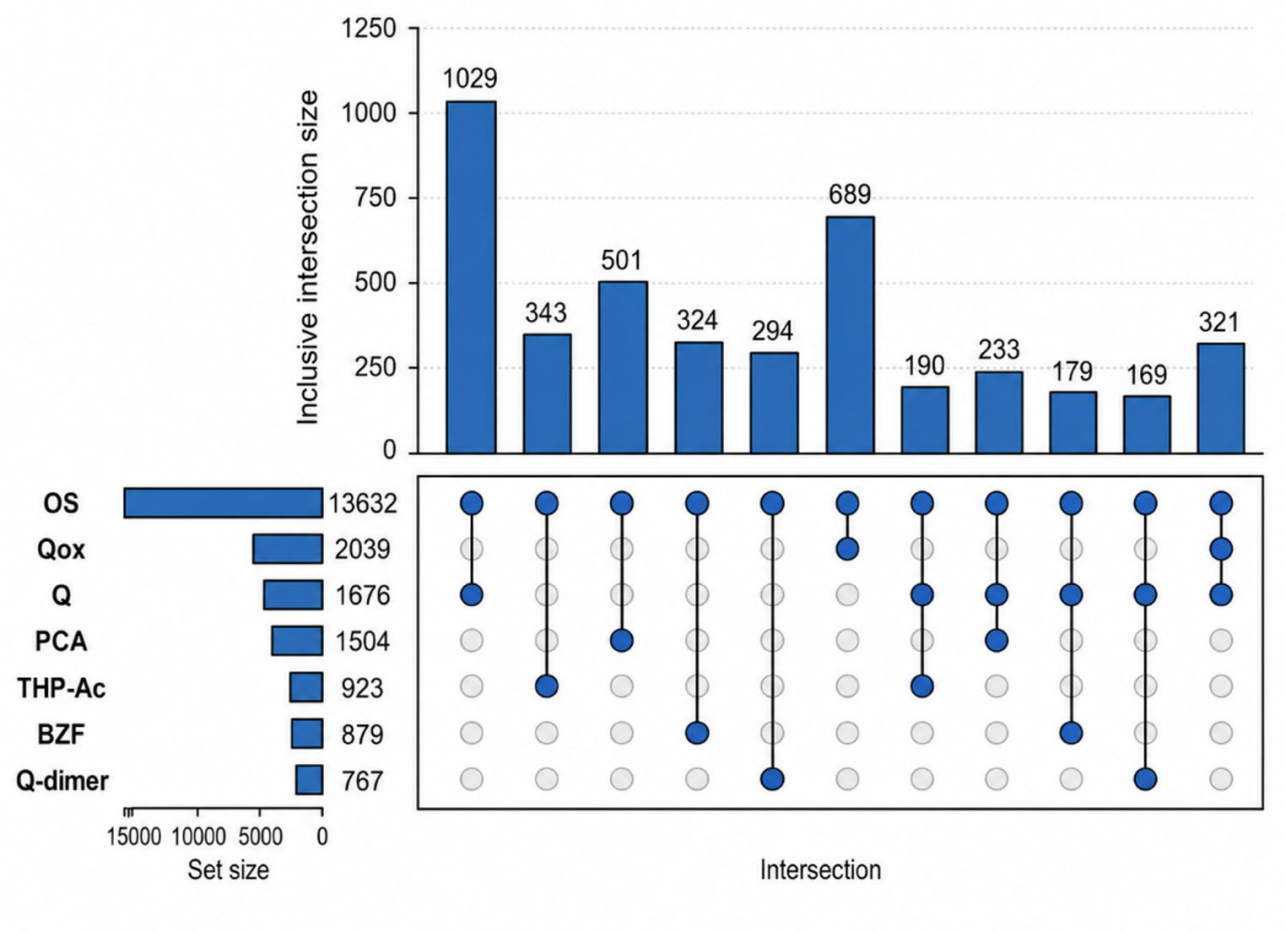
Upset plot depicting the common targets for quercetin (Q) and oxidative metabolites (Qox). The top bar graph showed the size of each group, while the matrix panel below denoted the contributing species: black filled circles indicated presence within the corresponding target set, and grey circles indicated absence

As shown in Fig. 4, integration of these datasets yielded a final Qox target pool comprising 2039 unique targets. The Q–OS target set contained 1029 targets, of which 321 were potentially modulated by Qox. Specifically, 190, 233, 179, and 169 targets were associated with THP-Ac, PCA, BZF, and the Q-dimer, respectively. Detailed information on the overlapping targets is provided in Supplementary Table S3.

#### Network involvement analysis

**Fig. 5:**
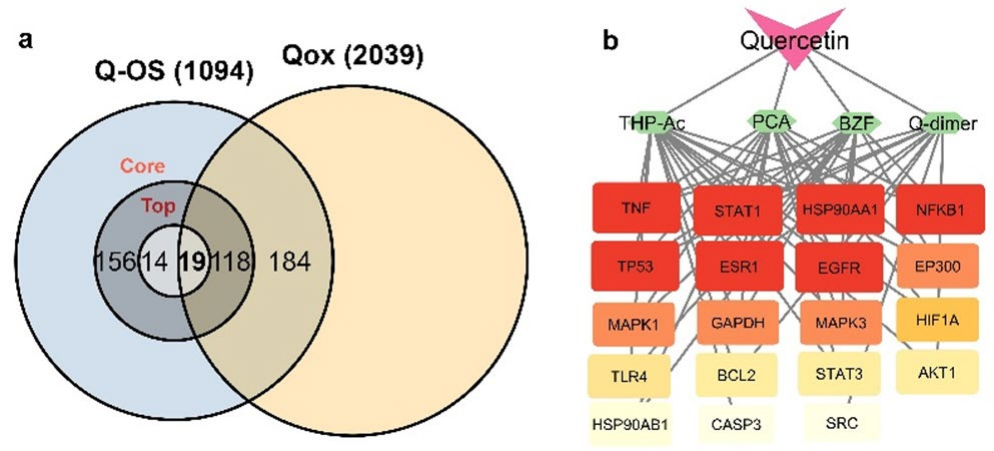
The roles of quercetin oxidative metabolites (Qox) within the protein–protein interaction (PPI) network of quercetin-mediated oxidative stress (Q-OS). **a** Venn diagram illustrating the target overlapping between Q-OS network and Qox. **b** Interaction network showing the connectivity of top targets associated with Qox, with red nodes representing targets of higher network centrality

To elucidate the roles of Qox within the Q-OS network, a well-defined network architecture of Q-OS was first built. All common targets of Q and OS were imported into STRING to generate a PPI network. Topological screening of this PPI network using six centrality algorithms identified 315 core targets. The top 10% highest-degree nodes within core set were further designated as “top targets”, yielding 33 prioritized targets. Among these, 137 core targets and 19 top targets showed potential associations with the Qox (Fig. 5a). To visualize this bio-crosslinked network, the core intersection targets set was further integrated with the Qox components to construct a compound–target interaction network (Supplementary Figure S3). A total of 90, 98, 84, and 74 targets were associated with THP-Ac, PCA, BZF, and Q-dimer, respectively. Detailed information on the intersection targets is provided in Supplementary Table S3. Consistently, within the 33 top targets, 17, 14, 13, and 11 were linked to THP-Ac, PCA, BZF, and Q-dimer, including representative stress-responsive and inflammatory regulators such as TNF, STAT1, HSP90AA1, NFKB1, ESR1, and EGFR among others (Fig. 5b), suggesting that Qox-mediated regulation may act through these key hubs, thereby exerting broader effects across the PPI network.

#### Target affinity analysis

To further evaluate the interactions between Qox and these top targets, molecular docking analyses were performed. Targets were firstly filtered based on the availability of high-resolution structural data and the presence of well-defined small-molecule binding pockets, such as kinase ATP sites, catalytic centers, or validated inhibitor-binding grooves [49]. A representative panel (8 proteins) was retained, including kinases (EGFR, MAPK1) [50], molecular chaperones (HSP90AA1, HSP90AB1) [51], to cancer cell survival (CASP3 [52], STAT3 [53], BCL2 [54], ESR1 [55]). The corresponding protein structures were retrieved from the PDB database with selected entries summarized in Supplementary Table S1.

**Fig. 6:**
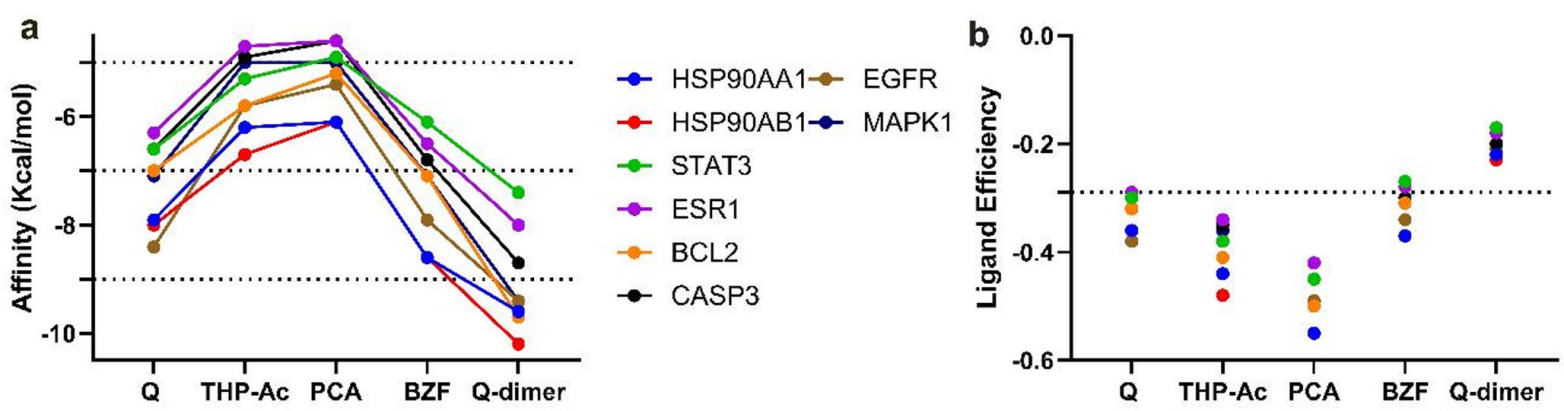
Molecular docking evaluation for quercetin (Q) and oxidative metabolites (THP-Ac, PCA, BZF, and the Q-dimer ) with selected top Q-OS targets. **a** Binding affinities, using –5.0 kcal/mol as the cutoff for meaningful binding. **b** Ligand efficiency, using 0.29 as the cutoff for qualified ligands

The binding affinity and ligand efficiency were evaluated to quantitatively characterize binding potential. We adopted the commonly used empirical interpretation ranges reported in large-scale docking benchmarking studies by Wong et al. [56], in which molecular docking simulations were systematically evaluated across hundreds of compounds and protein targets. In this study, binding energies lower than approximately − 7.0 kcal/mol were considered indicative of relatively favorable predicted interactions, whereas values between − 5.0 and − 7.0 kcal/mol were interpreted as reflecting moderate interaction tendencies. As shown in Fig. 6a, all selected targets were theoretically accessible to Qox. BZF and Q-dimer were exhibiting consistently stronger affinities than THP-Ac and PCA. Compared with Q, Q-dimer showed the more negative binding affinity across all targets. BZF exhibited more negative affinity for five targets (HSP90AA1, HSP90AB1, BCL2, ESR1, and CASP3), and comparable affinities toward MAPK1.

Schultes et al., in their study on fragment-based drug discovery and hit optimization, found that ligand efficiency values around 0.29 kcal mol⁻^1^ per heavy atom are generally associated with acceptable binding efficiency during early-stage lead optimization [57]. Following this work, ligand efficiency was additionally evaluated in the present work as a complementary empirical descriptor to partially reduce molecular size-related inflation of docking affinity scores. Only Q-dimer consistently remained below this benchmark for all targets (Fig. 6b). These observations suggest that the favorable docking energies predicted for Q-dimer may be partially influenced by its larger molecular framework and therefore should be interpreted cautiously.

**Fig. 7:**
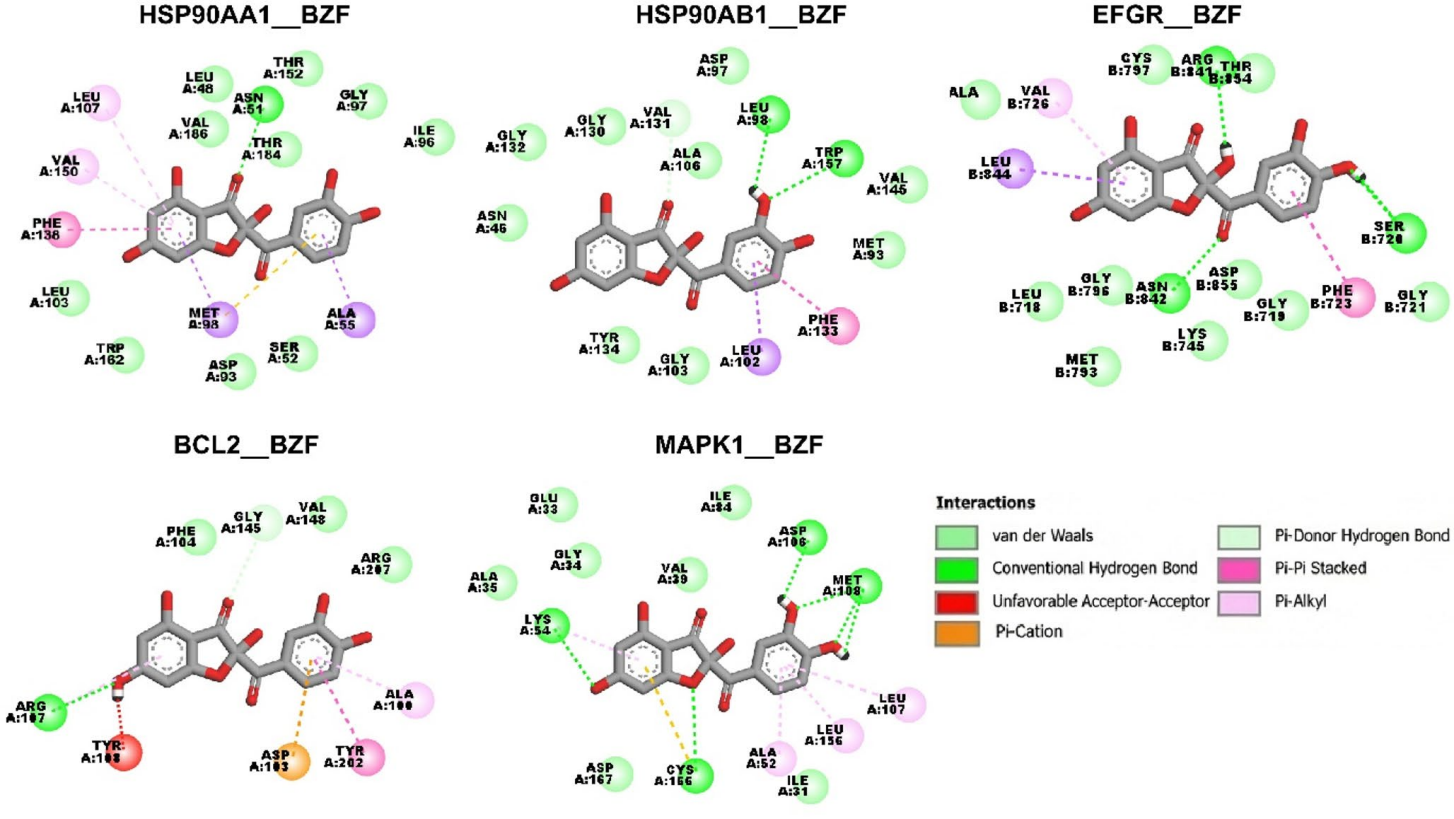
Predicted two-dimensional binding interaction profiles of BZF within biologically relevant ligand-binding pockets of selected protein targets. HSP90AA1 (PDB ID: 5CF0); HSP90AB1 (PDB ID: 6N8Y); EGFR (PDB ID: 8A27); BCL2 (PDB ID: 8HTS); MAPK1/ERK2 (PDB ID: 8AOJ)

Collectively, these findings suggest that Qox possesses substantial binding potential on top targets in regulatory network. Although Q-dimer demonstrated the most favorable affinities and Ki values, its low ligand efficiency. When all metrics were considered collectively, BZF emerged as the most promising Qox derivative, combining favorable predicted binding affinities with acceptable ligand efficiency, particularly toward HSP90AB1, HSP90AA1, EGFR, MAPK1, and BCL2 (binding scores ≤  − 7.0 kcal/mol). To further evaluate the biological relevance of these high predicted affinities, the corresponding BZF–protein complexes were subjected to detailed interaction analysis (Fig. 7). The results show that, in HSP90AA1, BZF occupied the N-terminal ATP/inhibitor-binding pocket and contacted Asp93 and Thr184, which are established residues involved in the hydrogen-bond network of HSP90 inhibitors within the nucleotide-binding cavity [58]. Similarly, in HSP90AB1, BZF strongly interacted with the conserved acidic residue Asp97, which is also located within the N-terminal ATP-binding pocket [59]. In EGFR, BZF occupied the kinase-domain inhibitor-binding region and interacted with Lys745 [60] and Cys79, two residues closely associated with catalytic function and the binding of clinically relevant EGFR inhibitors [61]. In BCL2, BZF contacted Phe104, Arg107, Gly145, and Val148 within the canonical BH3-binding groove, which mediates the recognition of pro-apoptotic BH3-domain proteins and is targeted by BCL2 inhibitors [62, 63]. BZF was also positioned within the ATP-binding pocket of ERK2, involving the conserved catalytic Lys54 and the hinge-region residue Met108 [64]. These results indicate that the predicted BZF poses were located within experimentally characterized functional or inhibitor-binding cavities rather than unrelated surface pockets.

### Functional enrichment analysis of oxidative metabolites

**Fig. 8:**
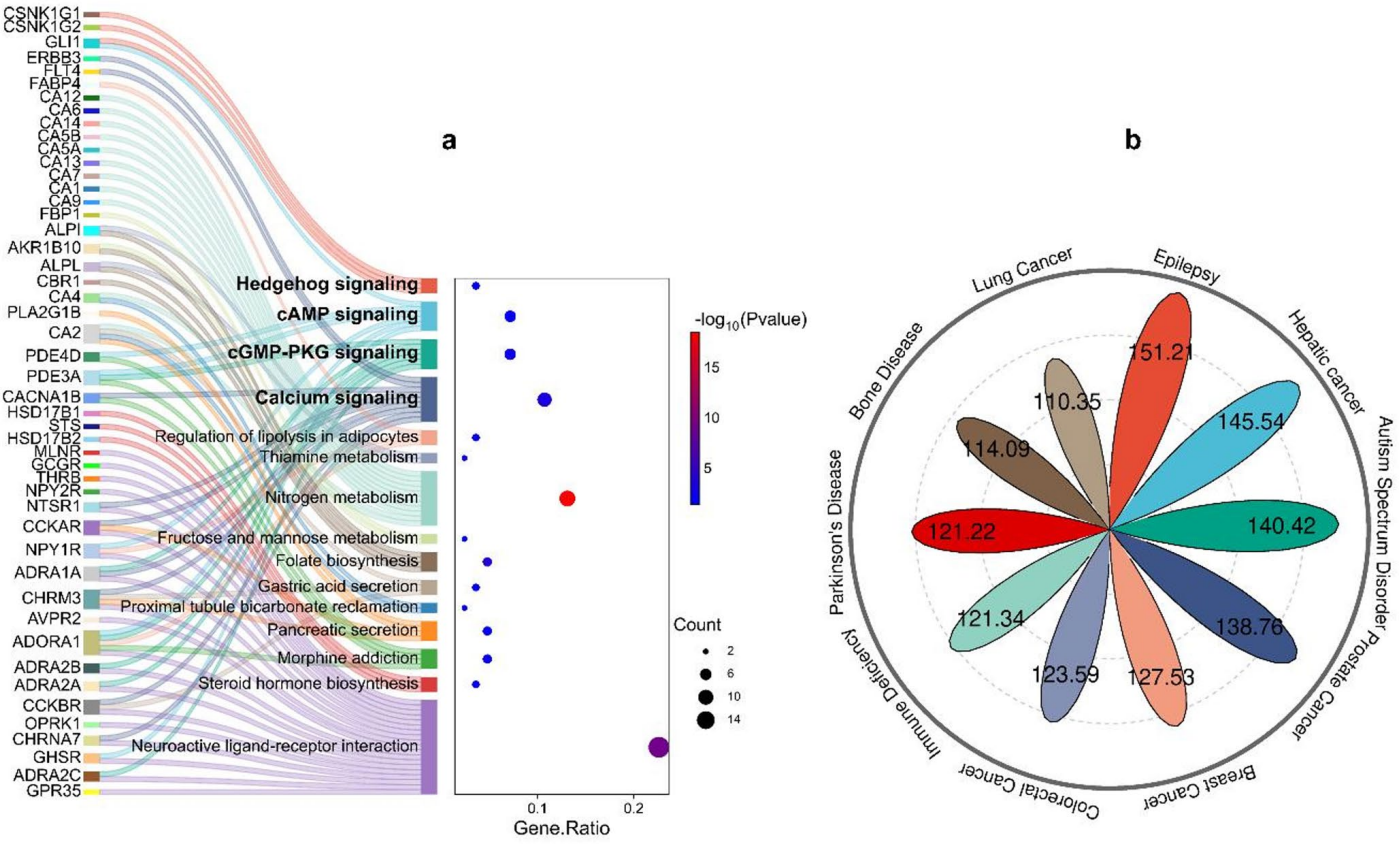
Pathway and disease enrichment of oxidative metabolites (Qox)-associated core targets within quercetin-mediated oxidative stress (Q-OS) network. **a** Sankey map showing enriched KEGG pathways (*p* < 0.05) and corresponding enrichment-assigned targets. **b** The flower diagram illustrating enriched score of related disease

To further elucidate the biological functions associated with Qox involvement, KEGG pathway enrichment analysis was performed on the common targets. A total of 195 pathways were significantly enriched (*p* < 0.05; Supplementary Table S4), including 16 pathways associated with the core targets (Fig. 8a). These results indicate that Qox predominantly acts across three different dimensions of redox-responsive signaling within the Q–OS network. The Hedgehog pathways [65], cAMP [66], cGMP–PKG [67], and enriched calcium [68] likely represent the core regulatory modules, as they are recognized ROS-sensing axes closely linked to the control of mitochondrial stability and inflammatory responses. Meanwhile, metabolic pathways such as folate, thiamine, fructose/mannose, nitrogen metabolism, and adipocyte lipolysis regulation support redox homeostasis by maintaining cofactor pools. Moreover, the enrichment of steroid hormone biosynthesis and neuroactive ligand–receptor interaction further reflected the regulation of endocrine. Detailed KEGG pathway enrichment results for individual compounds are provided in Supplementary Table S5.

Further disease-score analysis (Fig. 8b) showed pathological relevance of the Qox-responsive targets, with top associations observed across four major disease domains: neurological disorders (epilepsy, autism spectrum disorder, Parkinson’s disease), cancers (hepatic, prostate, breast, colorectal, and lung cancer), immune disorders (immune deficiency disease), and bone-related diseases. Detailed disease associations for individual compounds are provided in Supplementary Figure S4.

### Potential mechanism

**Fig. 9:**
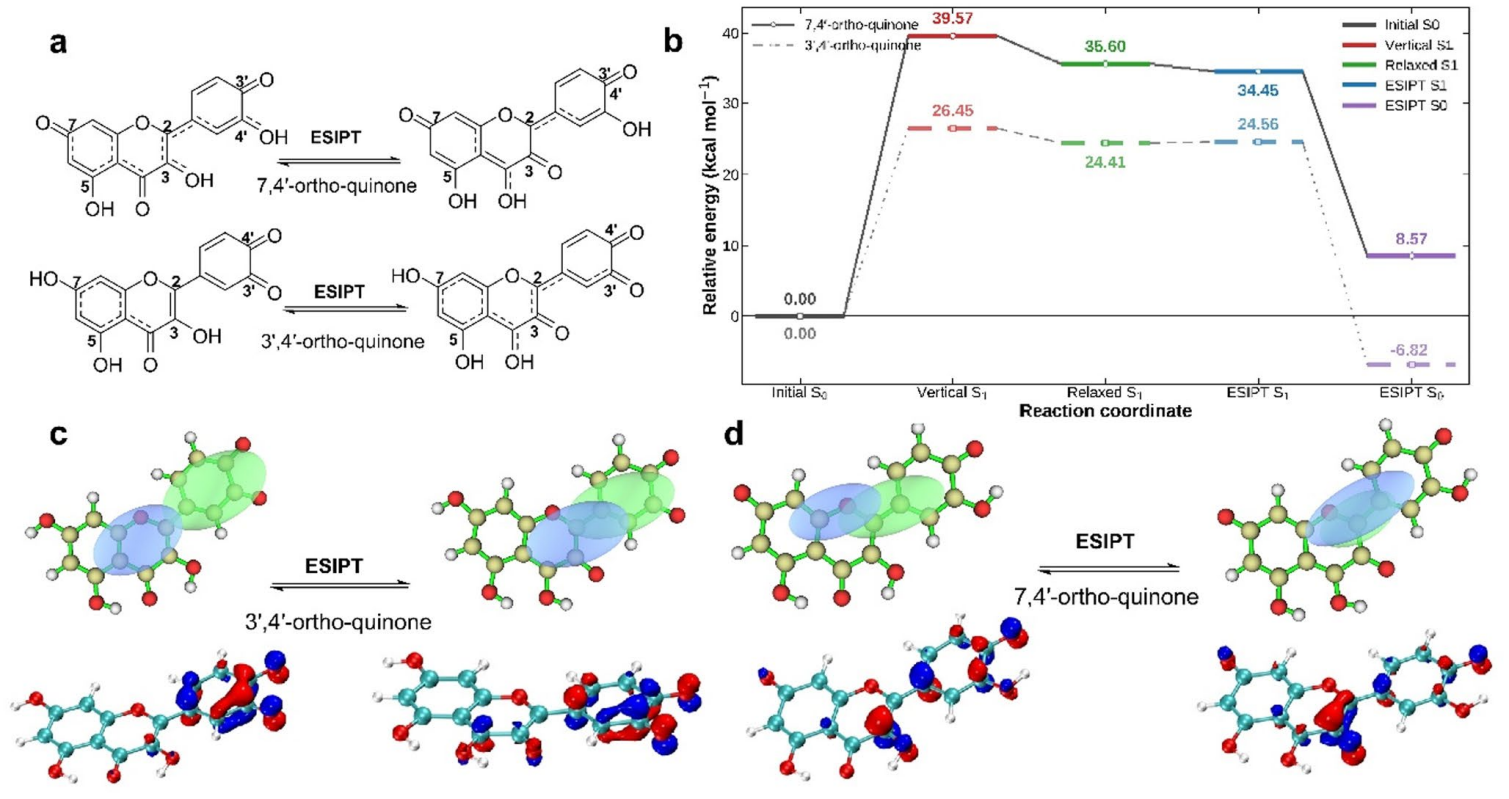
Time-dependent density functional theory (TD-DFT) analysis of the excitation–excited-state intramolecular proton-transfer–relaxation pathways of quercetin-derived quinone intermediates. **a** Four theoretically plausible quercetin quinone structures generated with or without excited-state intramolecular proton transfer. **b** Relative-energy profiles of the 7,4′-diquinone and 3′,4′-ortho-quinone along the initial ground state (S0), vertically excited first singlet state (vertical S1), relaxed first singlet state (relaxed S1), proton-transferred first singlet state (ESIPT S1), and proton-transferred ground state (ESIPT S0). Energies are reported in kcal mol⁻^1^ relative to the corresponding initial S0 structure. **c** Natural transition orbitals (NTOs) and lowest unoccupied molecular orbitals (LUMOs) of the 3′,4′-ortho-quinone before and after excited-state intramolecular proton transfer (ESIPT). **d** Corresponding NTOs and LUMOs of the 7,4′-diquinone before and after ESIPT. In the NTO plots, blue and green surfaces represent the hole and electron components, respectively. In the LUMO plots, red and blue surfaces denote opposite orbital phases

To elucidate the mechanistic basis underlying the unexpectedly potent reducing metabolites generated during the oxidation of Q, we conducted a systematic examination of its transient oxidative intermediates. The quinone scaffold was widely recognized as a key oxidative intermediate of flavonoids [69]; however, for Q, debate persisted over whether the primary quinone forms at the B-ring 3′,4′-ortho position or as a 7,4′-diquinone. Given the prevalence of excited-state intramolecular proton transfer (ESIPT) in flavonols [70], four plausible diquinone structures are theoretically feasible (Fig. 9a).

TD-DFT calculations were further performed to characterize the excitation–ESIPT–relaxation process for both diquinones. The vertical excitation energies from the ground state (S0) to the first excited state (S1) were 39.57 kcal mol⁻^1^ for the 7,4′-diquinone and 26.45 kcal mol⁻^1^ for the 3′,4′-ortho-quinone (Fig. 9b). Subsequent excited-state relaxation lowered these energies to 35.60 and 24.41 kcal mol⁻^1^, respectively. ESIPT was energetically favorable for the 7,4′-diquinone and nearly isoenergetic for the 3′,4′-ortho-quinone, indicating that proton transfer is feasible on the S1 potential-energy surface for both structures. Although a small energetic penalty was predicted for the latter, ultrafast spectroscopic studies have shown that ESIPT in Q can occur on a femtosecond timescale, potentially before complete excited-state relaxation [71].

NTO analysis was subsequently used to compare the excitation characteristics before and after ESIPT. For the 3′,4′-ortho-quinone, the hole and electron distributions remained broadly similar after proton transfer. Consistently, the LUMO distribution of the resulting ground-state structure remained largely unchanged and was predominantly localized over the B-ring conjugated region (Fig. 9c). In contrast, the 7,4′-diquinone exhibited a pronounced change in NTO distribution after ESIPT. Accordingly, the post-ESIPT 7,4′-diquinone exhibited a distinct LUMO distribution compared with the other three quinone structures, characterized by pronounced LUMO localization around the C2–C3 region (Fig. 9d). This localization rendered the C2–C3 highly susceptible to nucleophilic attack (e.g., by water), facilitating the engagement of external reducing equivalents, thereby accelerating the formation of metabolites with enhanced reducing capacity (e.g., BZF). This behavior was strongly dependent on the flavonol-specific ESIPT process, which may underline the exceptionally potent antioxidant performance of Q compared with other polyphenols.

## Discussion

Although Q occurs mainly as glycosides in plant-based foods [72], the aglycone generally exhibits greater antioxidant potency [73]. However, despite the efficient absorption of some glycosides, free Q remains at very low circulating concentrations due to rapid metabolism and conjugation [74]. This discrepancy between its strong intrinsic reactivity and limited systemic exposure highlights the need for further studies to clarify its antioxidant mechanism.

In this study, we propose that Q’s exceptional efficacy may be associated with a coordinated antioxidant network in which its direct and indirect actions are intrinsically interconnected. Rather than merely terminating radicals, Q’s directly scavenging reactions generated oxidative metabolites with heightened biological activity. These metabolites may further participate in subsequent radical-scavenging processes and could be associated with selected regulatory networks related to indirect antioxidant defenses. This hypothesis is grounded by accumulating experimental and theoretical evidence demonstrating that oxidative transformation of Q, including oxidation under radical-generating conditions [75], can enhance its antioxidant activity. Our work, attributes such amplification effects to defined oxidative metabolites.

In the present study, a DPPH-based chemical oxidation system was used to partially mimic the radical-mediated oxidation of Q and generate early-stage oxidation products. DPPH is widely used in antioxidant assays because its reduction by antioxidants causes a measurable decrease in absorbance [16]. In the present study, DPPH served not only as a reaction-monitoring probe but also as a controlled radical oxidant. Owing to its steric hindrance and relatively high stability, DPPH provides a reproducible and moderately reactive radical system suitable for inducing and monitoring the controlled oxidation of Q [32]. Multiple oxidation products were detected, and the metabolites selected for further investigation were those that had also been previously reported in other relevant oxidation systems. Specifically, among the identified products, PCA and THP-Ac have been widely reported as major metabolic products of Q, particularly following gut microbiota-mediated biotransformation [76]. Previous studies have also demonstrated that Q intake can increase circulating PCA levels and attenuate lipopolysaccharide (LPS)-induced reductions in transendothelial electrical resistance (TEER) [76, 77]. Although direct in vivo evidence for the remaining oxidation products is still limited, they have been consistently observed in enzymatic oxidation, radical-mediated oxidation, and auto-oxidation systems. Moreover, oxidative transformation of Q may be conserved across different oxidative environments, resulting in the formation of highly consistent oxidation products [77, 78]. Therefore, these oxidation products may serve as supporting indicators of biologically relevant oxidative metabolism. However, the compounds explored in the present study should be regarded as preliminary assignments of oxidation products rather than a comprehensive characterization. We focused on products previously reported in Q radical-oxidation studies to improve the reliability of the assignments. Definitive characterization will require orthogonal analytical approaches, including preparative isolation, authentic standards, two-dimensional NMR spectroscopy, high-resolution tandem mass spectrometry at multiple collision energies, and time-resolved LC–MS analysis. Future studies should also evaluate at least two reaction-mixture concentrations and generate kinetic curves to monitor product formation and consumption over time. Fraction collection followed by antioxidant testing would further allow the abundance of individual metabolites to be correlated with the activity of the oxidized mixture.

Our computational findings are well aligned with the central hypothesis. DFT simulations revealed theoretically enhanced reactivity for Qox relative to Q, characterized by favorable electronic and thermodynamic properties. Notably, BZF exhibited the smallest frontier orbital energy gap and consistently lower PA values, supporting a clear preference for the SPLET mechanism, which is widely recognized as the dominant antioxidant pathway in aqueous environments [79]. The present thermodynamic results apply primarily to near neutral and physiological conditions and should not be directly extrapolated to strongly acidic or alkaline environments. Because pH-dependent protonation can alter the relative preference for HAT, SPLET, and SET–PT pathways. Network pharmacology analysis further highlighted the significance of Qox within the antioxidant network. Importantly, although the current Qox-related targets and pathway enrichments were derived from database-based prediction, the resulting pathway–disease interconnection map shows strong external consistency with existing experimental and mechanistic evidence. In neurological disease models, Q has been reported to exert neuroprotective effects in epilepsy-related systems, where its activity has been linked to modulation of excitatory neurotransmission and ion channel–associated processes, including Ca^2^⁺-coupled signaling pathways [80]. In parallel, the nitric oxide (NO)–cGMP–PKG pathway has been shown to regulate neurotransmitter release and synaptic activity, whereas dysregulated cAMP signaling is associated with abnormal neuronal firing and excitability [81, 82]. Hedgehog signaling is closely associated with cancer cell proliferation and survival, and experimental studies have demonstrated that Q suppresses key Hedgehog pathway components, including Smoothened and Gli1, leading to reduced proliferation in cancer cell models [83]. Notably, our docking results further suggest that BZF exhibits stronger binding affinity toward HSP90AA1 and BCL2. In bone-related diseases, the expression of heat shock protein HSP90AA1 has been reported as a hallmark of joint disease in patients with knee osteoarthritis [84]. In parallel, agents that can upregulate BCL-2 expression or inhibit the apoptotic pathway are being investigated as potential therapeutic targets for OA treatment [85]. Collectively, these convergent lines of evidence suggest that Qox-derived metabolites may contribute to biologically relevant regulatory networks, providing a rationale for their further prioritization and targeted validation in disease-specific pathway contexts.

Nevertheless, it should be noted that these computational results are subject to inherent limitations. DFT-based descriptors are sensitive to the choice of functional, basis set, and solvation model, which may influence the quantitative outcomes. In addition, implicit solvation models cannot fully capture specific solute–solvent interactions and dynamic effects present in biological systems [86]. Moreover, antioxidant activity in vivo is governed by complex factors, including local microenvironment, competing reaction pathways, and biomolecular interactions, which are not fully represented in static quantum chemical calculations [87]. Therefore, while DFT provides valuable mechanistic insights, the reducing capacity inferred from computational analyses also requires further experimental validation, including established chemical radical-scavenging assays as well as biologically relevant [88].

Besides, network pharmacology provides probabilistic rather than definitive evidence of target engagement and does not fully capture context-dependent determinants of biological activity, such as compound bioavailability, intracellular exposure, metabolic conversion, cell-type specificity, and pathway crosstalk [89]. Similarly, molecular docking provides only static predictions of binding poses and affinities. It does not fully account for protein flexibility, solvent effects, ligand bioavailability, intracellular concentrations, or competition with endogenous ligands. Therefore, the present findings should be interpreted as a hypothesis-generating framework rather than conclusive mechanistic proof. Future work should prioritize systematic validation along multiple complementary levels. First, at the computational level, the robustness of the predicted compound–target relationships should be assessed by cross-database comparison, sensitivity analysis to target list composition, and orthogonal docking/ molecular dynamics-based binding stability evaluation for key targets. Second, at the biochemical and biophysical level, direct target engagement should be confirmed using binding assays and enzymatic or functional activity assays for prioritized proteins, thereby distinguishing true binders from network-derived false positives. Third, at the cellular level, disease-relevant models should be used to verify whether Qox metabolites (e.g., BZF, Q-dimer) induce pathway-consistent responses. In parallel, transcriptomic/proteomic profiling can be leveraged to evaluate whether the global expression signatures induced by individual metabolites align with the predicted pathway modules, thereby validating the network-level coherence. Ultimately, integrating these validation layers will enable refinement of the predicted compound–pathway–disease axis and facilitate the selection of the most promising oxidative metabolites for downstream translational development.

The detailed examination of the oxidative intermediates reveals that the paradoxical formation of metabolites with superior reducing capacity following radical-driven oxidation of Q originates from the unique 7,4′-diquinone intermediate formed after ESIPT. Owing to its sharply localized LUMO, this intermediate is highly susceptible to nucleophilic attack (e.g., by water), thereby providing an entry point for external energetic input that drives subsequent rearrangement and reduction, ultimately yielding metabolites with enhanced reducing ability. The ESIPT mechanism is structurally restricted to systems containing an enol moiety, and consistent with previous reports, only flavonols have been shown to generate analogous benzofuranone-type scaffolds [90]. However, the reaction processes and the stability of the resulting intermediates vary substantially among different flavonols and therefore further studies are warranted to clarify the mechanistic details [91].

Overall, this study highlights the importance of successive reactive intermediates, particularly BZF, consistent with recent report that it confers an approximately 200-fold enhancement in cytoprotective efficacy against indomethacin-induced oxidative stress in Hs68 fibroblasts and Caco-2 cells [45]. This insight is especially consequential given that Q’s clinical potential has long been constrained by its well-recognized low bioavailability, for which no broadly applicable solutions have yet been established [92].

More broadly, this metabolite-centered framework introduces a conceptual shift with significant implications for drug discovery. For intrinsically unstable natural compounds, strategically directing the formation of therapeutically advantageous metabolites, through formulation engineering, enzymatic pathway modulation, or co-administration strategies, may offer a powerful means to enhance pharmacological efficacy in disease-specific contexts.

## Conclusion

These findings revisit the antioxidant network of Q from a metabolite-centered perspective. Rather than functioning as separate direct and indirect pathways, direct radical scavenging by Q may initiate oxidative transformations that generate metabolites capable of further redox reactions. These metabolites may subsequently participate in additional scavenging processes and could be associated with selected regulatory pathways related to antioxidant responses. Collectively, these findings suggest that Q-derived oxidative metabolites may contribute to the overall antioxidant profile of Q, with BZF representing a potentially relevant mediator. Notably, the formation of such oxidative metabolites may be intrinsically linked to the flavonol-specific ESIPT process.

## Supplementary Information

Below is the link to the electronic supplementary material.

Supplementary file1.

## Data Availability

All data supporting the findings of this study are available within the article and its Supplementary Information files.
