# Supplementary material for: Oxidation-derived metabolites sustain the antioxidant network of quercetin
Source: J Comput Aided Mol Des. 2026 Jul 18;40(1):182. doi: 10.1007/s10822-026-00878-3 (PMC13380631; doi:10.1007/s10822-026-00878-3)
Supplement: Supplementary file 1 — Supplementary file1. [file 10822_2026_878_MOESM1_ESM.docx]

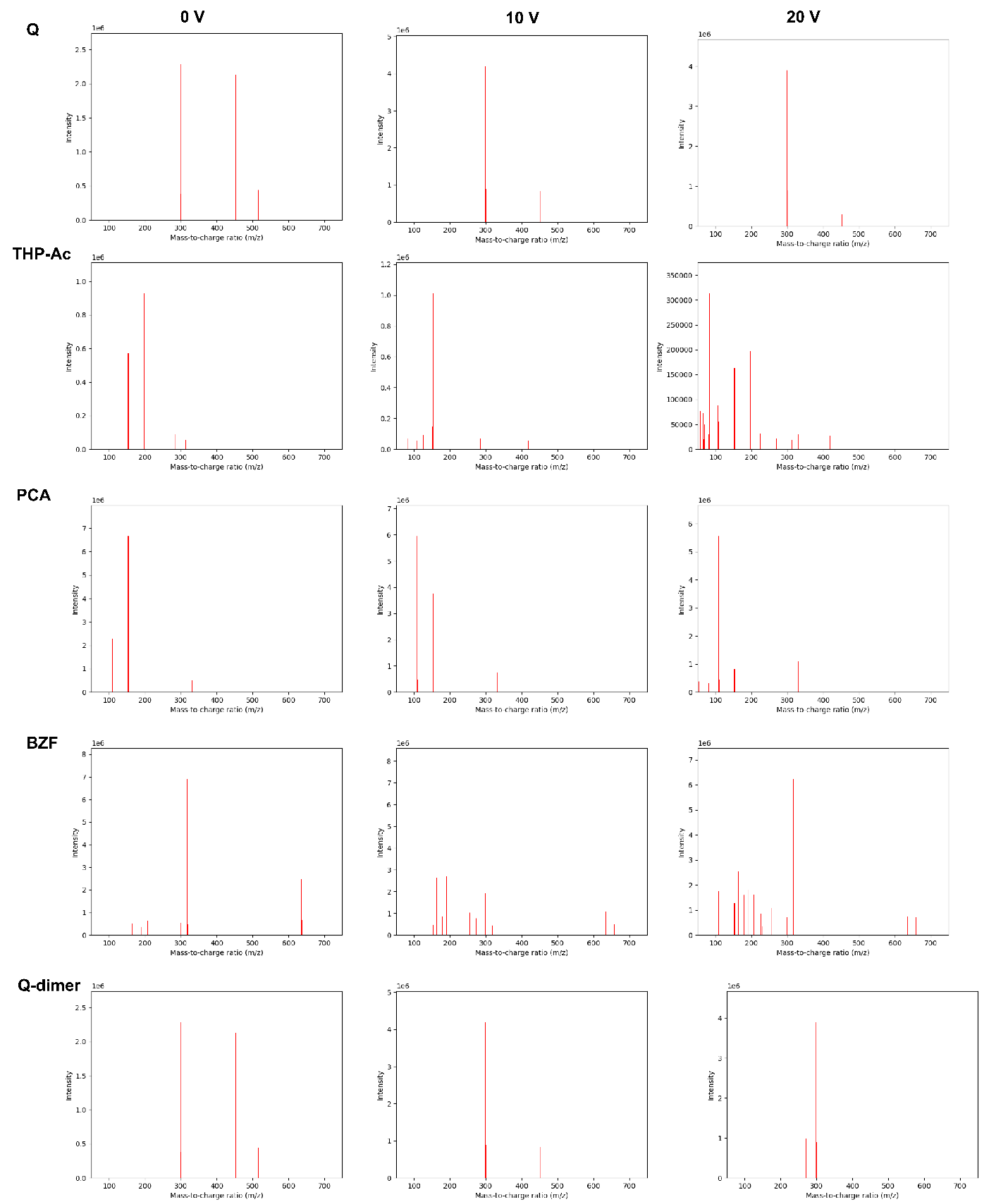


Supplementary Figure S1. MS/MS spectra for individual compounds acquired at collision energies of 0, 10, and 20 V.


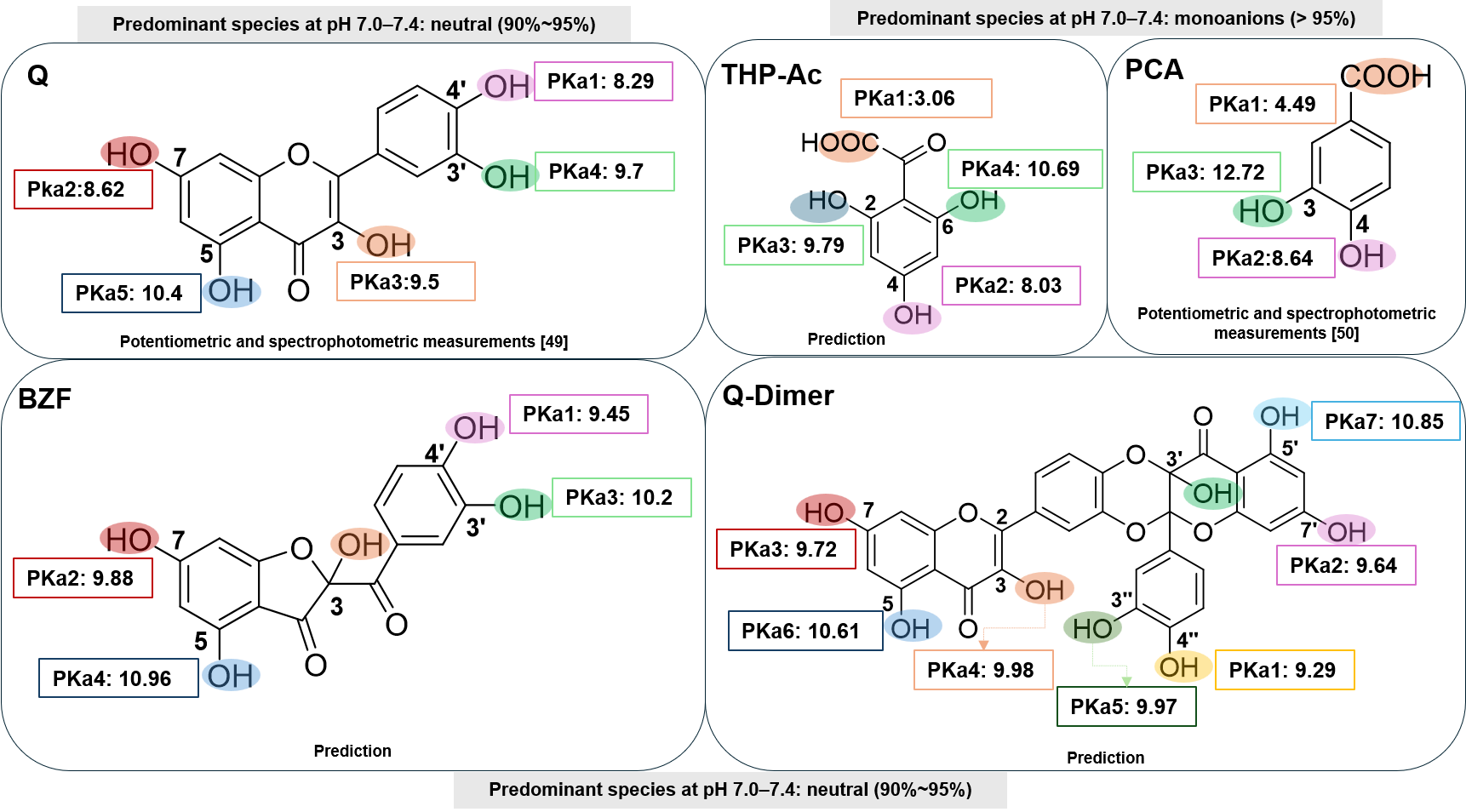


Figure S2. The relevant ionizable sites, pKa values, and predominant species at pH 7.0–7.4. Experimental pKa values were used when available, including those reported for quercetin [49] and protocatechuic acid [50]. For compounds without reliable experimental pKa data, site-specific pKa values were predicted using ACD/Labs Percepta pKa prediction. Predicted values that were clearly inconsistent with expected phenolic/carboxylic acid behavior were not used for site annotation.


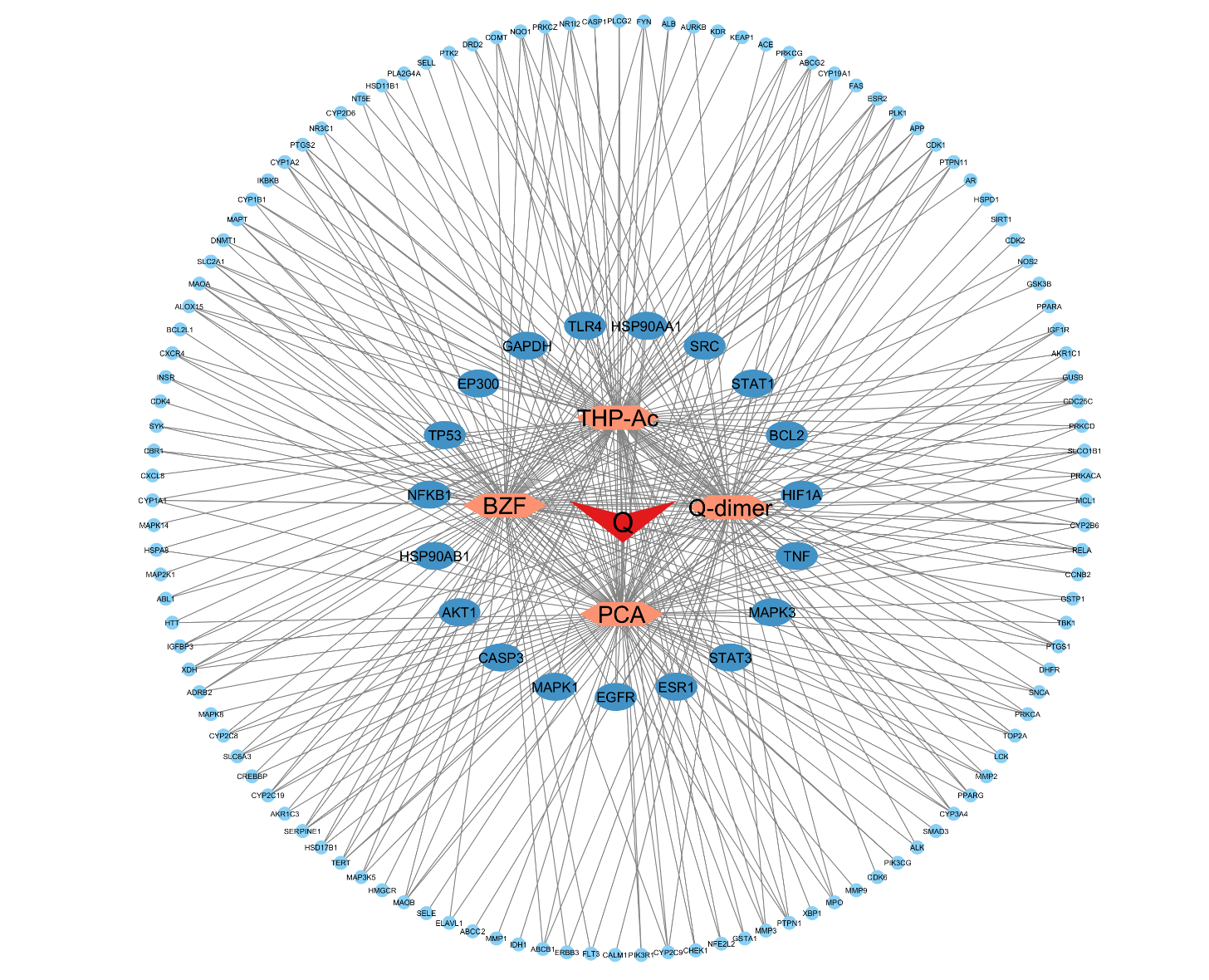


Supplementary Figure S3. Compound–target interaction network showing the connectivity of the core targets linked to Qox.


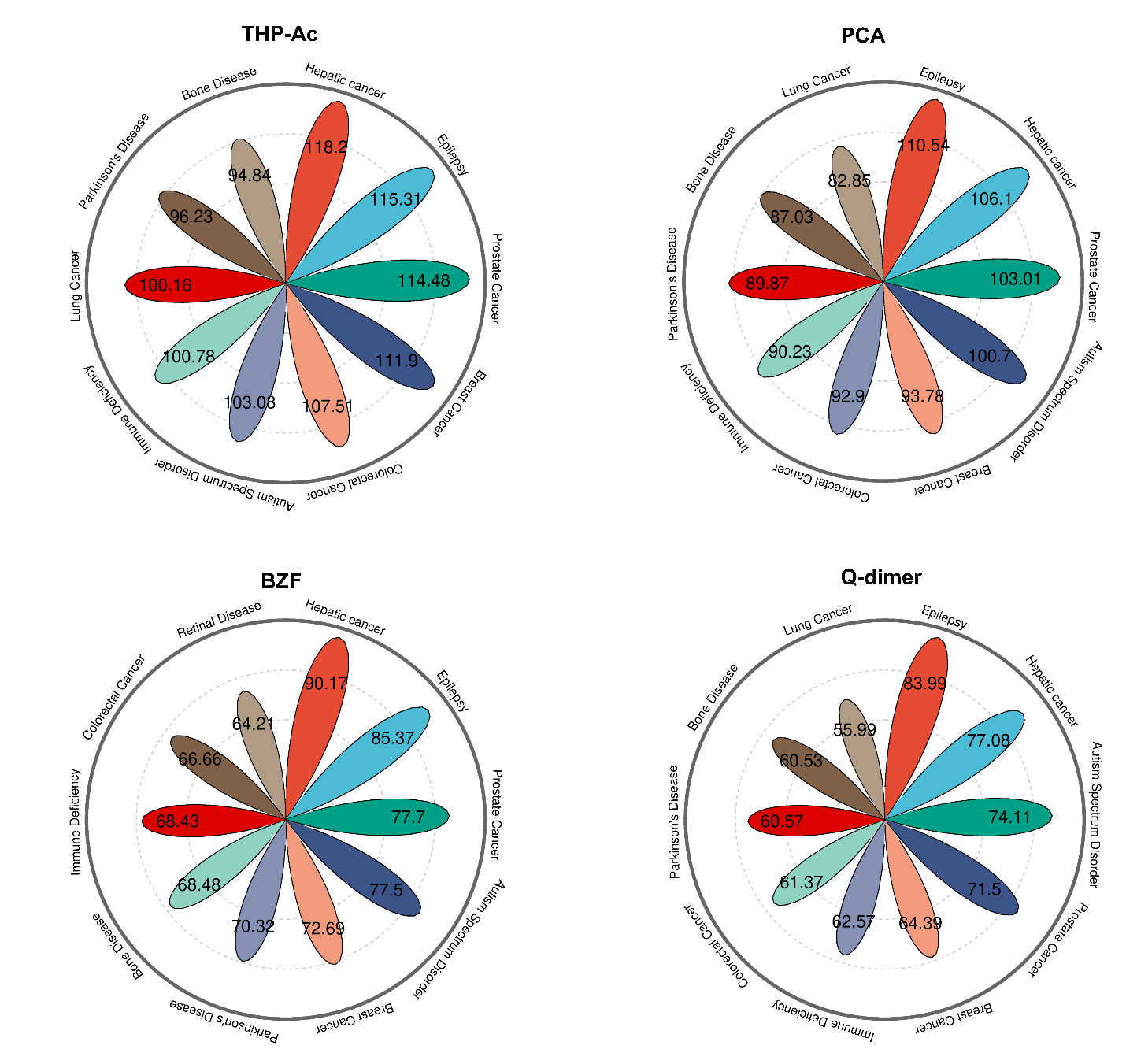


Supplementary Figure S4. Potential disease associations for individual metabolites.

Supplementary Table S1. Ligand-center coordinates, docking box sizes, and redocking RMSD values for validated protein targets.

| Protein | PDB ID | Ligand Center (x, y, z) | Box Size (Å) | RMSD (Å) |
| --- | --- | --- | --- | --- |
| EGFR | 4I24 | (-14.4, 6.14, -25.4) | 23 | 0.961 |
| MAPK1 | 8AOJ | (9.1, 10.3, 43.2) | 16 | 1.437 |
| CASP3 | 2XYG | (36.3, 28.6, 32.1) | 16 | 1.587 |
| HSP90AA1 | 5CFO | (-2.6, -9.8, -23.4) | 21 | 0.562 |
| HSP90AB1 | 6N8Y | (-3.8, -11.0, -23.5) | 19 | 0.994 |
| STAT3 | 6NJS | (13.0, 55.6, 0.3) | 33 | 1.62 |
| BCL2 | 8HTS | (-0.2, 9.4, 7.2) | 30 | 0.428 |
| ESR1 | 8DU8 | (17.2, 19.5, 23.6) | 24 | 0.86 |
| MAPK3 | 4QTB | (36.7, 54.8, 49.9) | 30 | 3.612 |
| AKT1 | 7NH5 | (-2.0, 16.0, -14.0) | 32 | 6.654 |
| AKT1 (alternative) | 3MVH | (-18.3, -2.4, 28.0) | 21 | Failed |
| SRC | Could not find suitable protein–ligand complex | | | |

Supplementary Table S2. Diagnostic ¹H and ¹³C NMR data of BZF in DMSO-d₆

| ¹H NMR | 3,4-disubstituted benzoyl moiety at δ_H_ 7.57 (d, J = 2.1 Hz, 1H), 7.56–7.53 (m, 1H), and 6.81 (d, J = 8.4 Hz, 1H). Additional signals at δ_H_ 5.96 (d, J = 1.4 Hz, 1H) and 5.91 (d, J = 1.4 Hz, 1H) corresponded to a trisubstituted benzoyl unit |
| --- | --- |
| ¹³C NMR | The spectrum revealed 15 carbon resonances in agreement with the proposed structure, including aromatic carbons at δ_C_ 171.96, 168.67, 158.72, 151.49, 144.83, 125.08, 123.90, 117.43, 115.00, 104.69, 100.55, and 96.71. Two carbonyl signals at δ_C_ 190.35 and 189.95 and a geminal-diol carbon at δ_C_ 90.42 further supported the structural assignment. |

Supplementary Table S3. Detailed information on the intersection targets.

| **Q-OS\|Qox** | **Q-OS\|THP-Ac** | **Q-OS\|PCA** | **Q-OS\|BZF** | **Q-OS\|Q-dimer** |
| --- | --- | --- | --- | --- |
| ABCB1 | ABCC1 | ABCB1 | ABCB1 | ABCB1 |
| ABCC1 | ABCG2 | ABCC1 | ABCC1 | ABCC1 |
| ABCC2 | ABL1 | ABCG2 | ABCG2 | ABCC2 |
| ABCG2 | ACHE | ACACA | ABL1 | ABCG2 |
| ABL1 | ACP1 | ACE | ACHE | ACACA |
| ACACA | ADRB1 | ACHE | ACP1 | ACHE |
| ACE | ADRB2 | ACP1 | ADORA1 | ACP1 |
| ACHE | AKR1A1 | ADRA2A | ADRB2 | ADORA1 |
| ACP1 | AKR1B1 | ADRA2C | AHR | ADRA1A |
| ADORA1 | AKR1B10 | ADRB1 | AKR1A1 | ADRA2B |
| ADRA1A | AKR1C1 | ADRB2 | AKR1B1 | ADRA2C |
| ADRA2A | AKR1C2 | ADRB3 | AKR1B10 | AKR1A1 |
| ADRA2B | AKR1C3 | AGTR1 | AKR1C2 | AKR1B1 |
| ADRA2C | AKT1 | AHR | ALOX12 | AKR1B10 |
| ADRB1 | ALB | AKR1B1 | ALOX15 | AKT1 |
| ADRB2 | ALOX12 | AKR1B10 | ALOX5 | ALK |
| ADRB3 | ALOX15 | AKR1C1 | ALPI | ALOX12 |
| AGTR1 | ALOX5 | AKR1C2 | ALPL | ALOX15 |
| AHR | ANPEP | AKR1C3 | APAF1 | ALOX5 |
| AKR1A1 | APEX1 | ALB | APEX1 | ALPI |
| AKR1B1 | APP | ALK | APP | APEX1 |
| AKR1B10 | BCHE | ALOX12 | AR | AURKB |
| AKR1C1 | BCL2 | ALOX15 | AURKB | AVPR2 |
| AKR1C2 | BLM | ALOX5 | AVPR2 | BACE1 |
| AKR1C3 | CA1 | ALPL | BACE1 | BCL2 |
| AKT1 | CA12 | ANPEP | CA1 | CA1 |
| ALB | CA13 | APEX1 | CA12 | CA12 |
| ALK | CA14 | APP | CA13 | CA13 |
| ALOX12 | CA2 | AVPR2 | CA14 | CA14 |
| ALOX15 | CA3 | BCHE | CA2 | CA2 |
| ALOX5 | CA4 | BCL2L1 | CA3 | CA3 |
| ALPI | CA5A | BLM | CA4 | CA4 |
| ALPL | CA5B | CA1 | CA5A | CA5A |
| ANPEP | CA6 | CA12 | CA6 | CA6 |
| APAF1 | CA7 | CA13 | CA7 | CA7 |
| APEX1 | CA9 | CA14 | CA9 | CACNA1B |
| APP | CACNA1C | CA2 | CACNA1B | CALM1 |
| AR | CAMK4 | CA3 | CAMK2A | CBR1 |
| AURKB | CASP1 | CA4 | CAMK4 | CCNB2 |
| AVPR2 | CASP2 | CA5A | CAMKK2 | CDC25C |
| BACE1 | CASP3 | CA5B | CBR1 | CDK1 |
| BCHE | CCKBR | CA6 | CCNB2 | CDK2 |
| BCL2 | CDC25C | CA7 | CDC25C | CDK5 |
| BCL2L1 | CDK1 | CA9 | CDK1 | CDK6 |
| BLM | CDK5 | CACNA1B | CDK4 | CFTR |
| CA1 | CFTR | CACNA1C | CDK5 | CHRM1 |
| CA12 | CHEK1 | CAMK4 | CFTR | CHRNA7 |
| CA13 | CHRM3 | CAMKK2 | CHRM2 | CLK1 |
| CA14 | CHRNA7 | CASP1 | CLK1 | CLK2 |
| CA2 | COMT | CCKAR | CLK3 | CNR1 |
| CA3 | CREBBP | CCKBR | COMT | CSNK2B |
| CA4 | CSNK1G1 | CDK1 | CSNK1G1 | CTSD |
| CA5A | CSNK1G2 | CDK5 | CSNK1G2 | CXCR1 |
| CA5B | CSNK2B | CFTR | CSNK2B | CXCR4 |
| CA6 | CTSD | CHEK1 | CTSD | CYP19A1 |
| CA7 | CXCL8 | CHRM1 | CXCR1 | CYP1A1 |
| CA9 | CXCR1 | CHRM2 | CXCR4 | CYP1A2 |
| CACNA1B | CYP19A1 | CHRM3 | CYP19A1 | CYP1B1 |
| CACNA1C | CYP1A1 | CHRNA7 | CYP1A1 | CYP2B6 |
| CALM1 | CYP1A2 | CLK3 | CYP1A2 | CYP2C19 |
| CAMK2A | CYP1B1 | COMT | CYP1B1 | CYP2C8 |
| CAMK4 | CYP2B6 | CREBBP | CYP2B6 | CYP2C9 |
| CAMKK2 | CYP2C19 | CSNK1G1 | CYP2C19 | CYP2D6 |
| CASP1 | CYP2C9 | CSNK1G2 | CYP2C8 | CYP3A4 |
| CASP2 | CYP2J2 | CSNK2A1 | CYP2C9 | DAPK1 |
| CASP3 | CYP3A4 | CSNK2A2 | CYP2J2 | DRD2 |
| CBR1 | DAPK3 | CSNK2B | CYP3A4 | DRD3 |
| CCKAR | DHFR | CTSD | DAP | DRD4 |
| CCKBR | DNMT1 | CXCR1 | DRD1 | EDNRA |
| CCNB2 | DRD2 | CXCR4 | DRD4 | EDNRB |
| CDC25C | EDNRA | CYP19A1 | EDNRA | EGFR |
| CDK1 | EGFR | CYP1A1 | EGFR | ELAVL1 |
| CDK2 | EP300 | CYP1A2 | ELAVL1 | ERBB3 |
| CDK4 | ERN1 | CYP2B6 | EP300 | ERG |
| CDK5 | ESR1 | CYP2C19 | ERBB3 | ERN1 |
| CDK6 | ESR2 | CYP2C8 | ESR1 | ESR1 |
| CFTR | ESRRB | CYP2C9 | ESR2 | ESR2 |
| CHEK1 | FABP4 | CYP2J2 | ESRRB | ESRRB |
| CHRM1 | FAS | CYP3A4 | F2 | F2 |
| CHRM2 | FASN | DAP | FLT3 | FLT3 |
| CHRM3 | FBP1 | DAPK1 | FYN | FYN |
| CHRNA7 | FYN | DAPK3 | GAPDH | GHSR |
| CLK1 | GAPDH | DBH | GBA1 | GLI1 |
| CLK2 | GCGR | DNMT1 | GC | GLO1 |
| CLK3 | GLI1 | DRD1 | GHSR | GLS |
| CNR1 | GLS | EDNRA | GLI1 | GUSB |
| COMT | GRIN1 | EGFR | GLO1 | HAP1 |
| CREBBP | GSTA1 | ELANE | GLS | HIF1A |
| CSNK1G1 | GUSB | EP300 | GSK3B | HSD11B1 |
| CSNK1G2 | HAP1 | ERN1 | GSTP1 | HSD17B10 |
| CSNK2A1 | HIF1A | ESR1 | GUSB | HSD17B2 |
| CSNK2A2 | HMGCR | ESR2 | HAP1 | HSP90AA1 |
| CSNK2B | HSD17B1 | ESRRB | HDAC9 | HSPA1A |
| CTSD | HSD17B10 | FABP4 | HSD17B1 | HTR2C |
| CXCL8 | HSD17B2 | FBP1 | HSD17B10 | IGF1R |
| CXCR1 | HSF1 | FLT4 | HSD17B2 | KCNH2 |
| CXCR4 | HSP90AA1 | FYN | HSP90AA1 | KDM1A |
| CYP19A1 | HSPA1A | GAPDH | HSP90AB1 | LPO |
| CYP1A1 | HSPA8 | GLI1 | HSPA1A | MAOA |
| CYP1A2 | HTR3A | GLS | HTR2B | MAOB |
| CYP1B1 | HTT | GSTA1 | IGF1R | MAP2K2 |
| CYP2B6 | IGFBP3 | GSTP1 | IGFBP3 | MAPKAPK2 |
| CYP2C19 | IKBKB | GUSB | INSR | MAPT |
| CYP2C8 | KDM1A | HAP1 | KDM1A | MCL1 |
| CYP2C9 | LCK | HIF1A | KDR | MIF |
| CYP2D6 | MAOA | HIF1AN | KEAP1 | MMP12 |
| CYP2J2 | MAOB | HRH2 | LPO | MMP3 |
| CYP3A4 | MAP2K1 | HSD11B1 | MAOA | MPG |
| DAP | MAP2K2 | HSD17B1 | MAOB | MPO |
| DAPK1 | MAP3K5 | HSD17B10 | MAP2K2 | NEK6 |
| DAPK3 | MAPK1 | HSD17B2 | MAP3K5 | NFKB1 |
| DBH | MAPK14 | HSF1 | MAPK1 | NLRP3 |
| DHFR | MAPK3 | HSP90AA1 | MAPK3 | NOS2 |
| DNMT1 | MAPK6 | HSP90AB1 | MAPKAPK2 | NOX4 |
| DRD1 | MAPT | HSPA1A | MAPT | NQO1 |
| DRD2 | MCL1 | HSPA8 | MCL1 | NR1I2 |
| DRD3 | MIF | HSPD1 | MIF | NR3C1 |
| DRD4 | MLNR | HTT | MMP2 | NR4A1 |
| EDNRA | MMP12 | IDH1 | NEK6 | NT5E |
| EDNRB | MMP2 | IGF1R | NFKB1 | NUAK1 |
| EGFR | MMP3 | IGFBP3 | NOS2 | ODC1 |
| ELANE | MPG | INSR | NOX4 | OPRD1 |
| ELAVL1 | MPO | KDM1A | NQO1 | PAK1 |
| EP300 | NEK6 | KDM4A | NR1I2 | PDE3A |
| ERBB3 | NFE2L2 | LCK | NR3C1 | PDE5A |
| ERG | NFKB1 | MAOA | NR4A1 | PFKFB3 |
| ERN1 | NOX4 | MAOB | NT5E | PIK3C2G |
| ESR1 | NQO1 | MAP2K2 | NUAK1 | PIM1 |
| ESR2 | NQO2 | MAP3K5 | ODC1 | PKN1 |
| ESRRB | NR1H2 | MAPK1 | PAK1 | PKN2 |
| F2 | NR1H3 | MAPK3 | PDE3A | PLA2G2A |
| FABP4 | NR1I2 | MAPK6 | PDE5A | PLAT |
| FAS | NR4A1 | MAPK8 | PFKFB3 | PLK1 |
| FASN | NTSR1 | MAPT | PIK3C2G | PLK4 |
| FBP1 | ODC1 | MCL1 | PIK3CG | PPARG |
| FLT3 | OPRK1 | MIF | PIM1 | PRKCZ |
| FLT4 | PCNA | MMP1 | PKN1 | PTGS1 |
| FYN | PDE3A | MMP13 | PLA2G1B | PTGS2 |
| GAPDH | PDE5A | MMP2 | PLK1 | PTK2 |
| GBA1 | PDK1 | MMP3 | PLK4 | PTPN1 |
| GC | PDPK1 | MMP9 | PRKACA | PTPN11 |
| GCGR | PIK3C2G | MPG | PRKCA | RELA |
| GHSR | PIK3R1 | MPO | PRKCD | RPS6KA3 |
| GLI1 | PIN1 | NAT1 | PRKCG | SERPINE1 |
| GLO1 | PLA2G1B | NEK6 | PRKCH | SHBG |
| GLS | PLA2G2A | NFE2L2 | PRKCZ | SLC16A7 |
| GRIN1 | PLCG2 | NFKB1 | PRKD3 | SLC22A6 |
| GSK3B | PLK1 | NOX1 | PSMB5 | SLC2A1 |
| GSTA1 | PPARG | NOX4 | PTGS1 | SLC6A3 |
| GSTP1 | PREP | NPY1R | PTGS2 | SLC6A5 |
| GUSB | PRKACA | NPY2R | PTPN1 | SLCO1B1 |
| HAP1 | PRKCA | NQO1 | PTPN11 | SLCO1B3 |
| HDAC9 | PRKCD | NQO2 | RELA | SRC |
| HIF1A | PRKCG | NR1H2 | RPS6KA3 | STAT1 |
| HIF1AN | PRKCH | NR1H3 | SERPINE1 | STS |
| HMGCR | PRKCI | NR1I2 | SHBG | SYK |
| HRH2 | PRKCZ | NR4A1 | SLC16A7 | TBK1 |
| HSD11B1 | PTGS1 | NTSR1 | SLC22A6 | TBXA2R |
| HSD17B1 | PTGS2 | ODC1 | SLC2A1 | TDP1 |
| HSD17B10 | PTPN1 | OPRD1 | SLC6A3 | TERT |
| HSD17B2 | PTPN11 | OPRK1 | SLC6A5 | TNF |
| HSF1 | RELA | OSR1 | SLCO1B1 | TOP1 |
| HSP90AA1 | SERPINE1 | OXSR1 | SLCO1B3 | TOP2A |
| HSP90AB1 | SF3B3 | PCNA | SMAD3 | TP53 |
| HSPA1A | SHBG | PDE4D | SNCA | TTR |
| HSPA8 | SLC22A6 | PDE5A | STAT1 | VEGFA |
| HSPD1 | SLC2A1 | PDK1 | SYK | VRK2 |
| HTR2B | SLC6A5 | PDPK1 | TDP1 | XDH |
| HTR2C | SLCO1B1 | PFKFB3 | TERT | YES1 |
| HTR3A | SLCO1B3 | PIK3C2G | THRB |  |
| HTT | SNCA | PIK3CG | TLR4 |  |
| IDH1 | SREBF2 | PIK3R1 | TNF |  |
| IGF1R | STAT1 | PIM1 | TOP1 |  |
| IGFBP3 | STAT3 | PIN1 | TOP2A |  |
| IKBKB | STS | PLA2G2A | TP53 |  |
| INSR | TBXA2R | PLA2G4A | TTR |  |
| KCNH2 | TDP1 | PLAT | TYR |  |
| KDM1A | TERT | PLAU | VRK2 |  |
| KDM4A | THRB | PLCG2 | XDH |  |
| KDR | TLR4 | PLK1 |  |  |
| KEAP1 | TNF | POLB |  |  |
| LCK | TOP1 | POLI |  |  |
| LPO | TOP2A | PPARA |  |  |
| MAOA | TP53 | PPARD |  |  |
| MAOB | TTR | PPARG |  |  |
| MAP2K1 | VEGFA | PRKACA |  |  |
| MAP2K2 | VRK2 | PRKCA |  |  |
| MAP3K5 | WEE1 | PRKCD |  |  |
| MAPK1 | XBP1 | PRKCG |  |  |
| MAPK14 | XDH | PRKCH |  |  |
| MAPK3 |  | PRKCI |  |  |
| MAPK6 |  | PRKCZ |  |  |
| MAPK8 |  | PSEN1 |  |  |
| MAPT |  | PTGS2 |  |  |
| MCL1 |  | PTK2 |  |  |
| MIF |  | PTK2B |  |  |
| MLNR |  | PTPN1 |  |  |
| MMP1 |  | PTPN11 |  |  |
| MMP12 |  | RELA |  |  |
| MMP13 |  | SELE |  |  |
| MMP2 |  | SELL |  |  |
| MMP3 |  | SERPINE1 |  |  |
| MMP9 |  | SFRP1 |  |  |
| MPG |  | SGK1 |  |  |
| MPO |  | SHBG |  |  |
| NAT1 |  | SIRT1 |  |  |
| NEK6 |  | SLC22A6 |  |  |
| NFE2L2 |  | SLC29A1 |  |  |
| NFKB1 |  | SLC2A1 |  |  |
| NLRP3 |  | SLC6A2 |  |  |
| NOS2 |  | SLC6A3 |  |  |
| NOX1 |  | SLC6A5 |  |  |
| NOX4 |  | SLCO1B1 |  |  |
| NPY1R |  | SLCO1B3 |  |  |
| NPY2R |  | SNCA |  |  |
| NQO1 |  | SREBF2 |  |  |
| NQO2 |  | STAT1 |  |  |
| NR1H2 |  | STAT3 |  |  |
| NR1H3 |  | STS |  |  |
| NR1I2 |  | TBK1 |  |  |
| NR3C1 |  | TBXA2R |  |  |
| NR4A1 |  | TDP1 |  |  |
| NT5E |  | TERT |  |  |
| NTSR1 |  | THRB |  |  |
| NUAK1 |  | TNF |  |  |
| ODC1 |  | TOP1 |  |  |
| OPRD1 |  | TOP2A |  |  |
| OPRK1 |  | TP53 |  |  |
| OSR1 |  | TTR |  |  |
| OXSR1 |  | TYR |  |  |
| PAK1 |  | WEE1 |  |  |
| PCNA |  | XDH |  |  |
| PDE3A |  |  |  |  |
| PDE4D |  |  |  |  |
| PDE5A |  |  |  |  |
| PDK1 |  |  |  |  |
| PDPK1 |  |  |  |  |
| PFKFB3 |  |  |  |  |
| PIK3C2G |  |  |  |  |
| PIK3CG |  |  |  |  |
| PIK3R1 |  |  |  |  |
| PIM1 |  |  |  |  |
| PIN1 |  |  |  |  |
| PKN1 |  |  |  |  |
| PKN2 |  |  |  |  |
| PLA2G1B |  |  |  |  |
| PLA2G2A |  |  |  |  |
| PLA2G4A |  |  |  |  |
| PLAT |  |  |  |  |
| PLAU |  |  |  |  |
| PLCG2 |  |  |  |  |
| PLK1 |  |  |  |  |
| PLK4 |  |  |  |  |
| POLB |  |  |  |  |
| POLI |  |  |  |  |
| PPARA |  |  |  |  |
| PPARD |  |  |  |  |
| PPARG |  |  |  |  |
| PREP |  |  |  |  |
| PRKACA |  |  |  |  |
| PRKCA |  |  |  |  |
| PRKCD |  |  |  |  |
| PRKCG |  |  |  |  |
| PRKCH |  |  |  |  |
| PRKCI |  |  |  |  |
| PRKCZ |  |  |  |  |
| PRKD3 |  |  |  |  |
| PSEN1 |  |  |  |  |
| PSMB5 |  |  |  |  |
| PTGS1 |  |  |  |  |
| PTGS2 |  |  |  |  |
| PTK2 |  |  |  |  |
| PTK2B |  |  |  |  |
| PTPN1 |  |  |  |  |
| PTPN11 |  |  |  |  |
| RELA |  |  |  |  |
| RPS6KA3 |  |  |  |  |
| SELE |  |  |  |  |
| SELL |  |  |  |  |
| SERPINE1 |  |  |  |  |
| SF3B3 |  |  |  |  |
| SFRP1 |  |  |  |  |
| SGK1 |  |  |  |  |
| SHBG |  |  |  |  |
| SIRT1 |  |  |  |  |
| SLC16A7 |  |  |  |  |
| SLC22A6 |  |  |  |  |
| SLC29A1 |  |  |  |  |
| SLC2A1 |  |  |  |  |
| SLC6A2 |  |  |  |  |
| SLC6A3 |  |  |  |  |
| SLC6A5 |  |  |  |  |
| SLCO1B1 |  |  |  |  |
| SLCO1B3 |  |  |  |  |
| SMAD3 |  |  |  |  |
| SNCA |  |  |  |  |
| SRC |  |  |  |  |
| SREBF2 |  |  |  |  |
| STAT1 |  |  |  |  |
| STAT3 |  |  |  |  |
| STS |  |  |  |  |
| SYK |  |  |  |  |
| TBK1 |  |  |  |  |
| TBXA2R |  |  |  |  |
| TDP1 |  |  |  |  |
| TERT |  |  |  |  |
| THRB |  |  |  |  |
| TLR4 |  |  |  |  |
| TNF |  |  |  |  |
| TOP1 |  |  |  |  |
| TOP2A |  |  |  |  |
| TP53 |  |  |  |  |
| TTR |  |  |  |  |
| TYR |  |  |  |  |
| VEGFA |  |  |  |  |
| VRK2 |  |  |  |  |
| WEE1 |  |  |  |  |
| XBP1 |  |  |  |  |
| XDH |  |  |  |  |
| YES1 |  |  |  |  |

Supplementary Table S4. Detailed KEGG pathway enrichment results for Qox.

| **Description** | **GeneRatio** | **BgRatio** | **pvalue** | **p.adjust** | **qvalue** |
| --- | --- | --- | --- | --- | --- |
| Lipid and atherosclerosis | 49/301 | 216/9396 | 1.30672E-28 | 3.80255E-26 | 1.32047E-26 |
| AGE-RAGE signaling pathway in diabetic complications | 28/301 | 101/9396 | 3.50722E-19 | 3.56858E-17 | 1.23923E-17 |
| Chemical carcinogenesis - reactive oxygen species | 40/301 | 227/9396 | 3.67895E-19 | 3.56858E-17 | 1.23923E-17 |
| HIF-1 signaling pathway | 28/301 | 110/9396 | 4.29258E-18 | 3.12285E-16 | 1.08444E-16 |
| Prostate cancer | 27/301 | 106/9396 | 1.70928E-17 | 9.94802E-16 | 3.45455E-16 |
| Hepatitis B | 32/301 | 163/9396 | 6.30127E-17 | 3.05611E-15 | 1.06127E-15 |
| Chemical carcinogenesis - receptor activation | 36/301 | 217/9396 | 1.89943E-16 | 7.89621E-15 | 2.74204E-15 |
| Fluid shear stress and atherosclerosis | 29/301 | 142/9396 | 6.44878E-16 | 2.34574E-14 | 8.14582E-15 |
| Serotonergic synapse | 26/301 | 115/9396 | 1.6226E-15 | 5.24641E-14 | 1.82187E-14 |
| Neurotrophin signaling pathway | 26/301 | 120/9396 | 4.87743E-15 | 1.30425E-13 | 4.52915E-14 |
| Nitrogen metabolism | 12/301 | 17/9396 | 5.04667E-15 | 1.30425E-13 | 4.52915E-14 |
| Calcium signaling pathway | 37/301 | 254/9396 | 5.37836E-15 | 1.30425E-13 | 4.52915E-14 |
| Proteoglycans in cancer | 33/301 | 204/9396 | 7.99945E-15 | 1.79065E-13 | 6.2182E-14 |
| Kaposi sarcoma-associated herpesvirus infection | 32/301 | 196/9396 | 1.60358E-14 | 3.33315E-13 | 1.15747E-13 |
| cAMP signaling pathway | 33/301 | 226/9396 | 1.65894E-13 | 3.21834E-12 | 1.1176E-12 |
| PD-L1 expression and PD-1 checkpoint pathway in cancer | 21/301 | 90/9396 | 4.78771E-13 | 8.70765E-12 | 3.02382E-12 |
| Bladder cancer | 15/301 | 41/9396 | 8.13144E-13 | 1.39191E-11 | 4.83355E-12 |
| Human immunodeficiency virus 1 infection | 31/301 | 213/9396 | 1.03507E-12 | 1.67337E-11 | 5.81094E-12 |
| Yersinia infection | 25/301 | 138/9396 | 1.24689E-12 | 1.90972E-11 | 6.63168E-12 |
| C-type lectin receptor signaling pathway | 22/301 | 105/9396 | 1.37101E-12 | 1.97039E-11 | 6.84239E-12 |
| Measles | 25/301 | 139/9396 | 1.47702E-12 | 1.97039E-11 | 6.84239E-12 |
| IL-17 signaling pathway | 21/301 | 95/9396 | 1.48965E-12 | 1.97039E-11 | 6.84239E-12 |
| VEGF signaling pathway | 17/301 | 60/9396 | 2.80559E-12 | 3.54968E-11 | 1.23266E-11 |
| Endocrine resistance | 21/301 | 99/9396 | 3.50065E-12 | 4.24453E-11 | 1.47396E-11 |
| Sphingolipid signaling pathway | 23/301 | 122/9396 | 4.32409E-12 | 5.03324E-11 | 1.74784E-11 |
| EGFR tyrosine kinase inhibitor resistance | 19/301 | 80/9396 | 4.55514E-12 | 5.09825E-11 | 1.77042E-11 |
| MAPK signaling pathway | 36/301 | 300/9396 | 5.0463E-12 | 5.3384E-11 | 1.85381E-11 |
| Human cytomegalovirus infection | 31/301 | 226/9396 | 5.13661E-12 | 5.3384E-11 | 1.85381E-11 |
| Neuroactive ligand signaling | 29/301 | 199/9396 | 5.63592E-12 | 5.65535E-11 | 1.96388E-11 |
| Alzheimer disease | 41/301 | 391/9396 | 1.24168E-11 | 1.20443E-10 | 4.18251E-11 |
| Pathways of neurodegeneration - multiple diseases | 46/301 | 483/9396 | 1.78968E-11 | 1.64326E-10 | 5.70638E-11 |
| PI3K-Akt signaling pathway | 39/301 | 362/9396 | 1.80702E-11 | 1.64326E-10 | 5.70638E-11 |
| Ras signaling pathway | 31/301 | 238/9396 | 2.02728E-11 | 1.78769E-10 | 6.20794E-11 |
| Pancreatic cancer | 18/301 | 77/9396 | 2.25506E-11 | 1.93006E-10 | 6.70233E-11 |
| FoxO signaling pathway | 23/301 | 133/9396 | 2.78248E-11 | 2.31343E-10 | 8.03362E-11 |
| Neuroactive ligand-receptor interaction | 39/301 | 370/9396 | 3.54051E-11 | 2.86191E-10 | 9.93828E-11 |
| Toxoplasmosis | 21/301 | 112/9396 | 4.2547E-11 | 3.34627E-10 | 1.16202E-10 |
| Apoptosis | 23/301 | 137/9396 | 5.21512E-11 | 3.99369E-10 | 1.38685E-10 |
| Hormone signaling | 29/301 | 219/9396 | 6.25199E-11 | 4.66495E-10 | 1.61995E-10 |
| Relaxin signaling pathway | 22/301 | 130/9396 | 1.183E-10 | 8.6063E-10 | 2.98862E-10 |
| ErbB signaling pathway | 18/301 | 86/9396 | 1.62053E-10 | 1.15018E-09 | 3.99411E-10 |
| Glioma | 17/301 | 76/9396 | 1.74084E-10 | 1.20615E-09 | 4.18849E-10 |
| Insulin resistance | 20/301 | 109/9396 | 1.88777E-10 | 1.27754E-09 | 4.43637E-10 |
| Hepatitis C | 24/301 | 159/9396 | 1.95942E-10 | 1.29589E-09 | 4.50011E-10 |
| Thyroid hormone signaling pathway | 21/301 | 122/9396 | 2.28345E-10 | 1.44453E-09 | 5.01627E-10 |
| Growth hormone synthesis, secretion and action | 21/301 | 122/9396 | 2.28345E-10 | 1.44453E-09 | 5.01627E-10 |
| Fc epsilon RI signaling pathway | 16/301 | 69/9396 | 3.41519E-10 | 2.11451E-09 | 7.34286E-10 |
| Prolactin signaling pathway | 16/301 | 71/9396 | 5.39299E-10 | 3.2695E-09 | 1.13537E-09 |
| Cholinergic synapse | 20/301 | 116/9396 | 6.02697E-10 | 3.57928E-09 | 1.24294E-09 |
| Arachidonic acid metabolism | 15/301 | 63/9396 | 8.27674E-10 | 4.78147E-09 | 1.66041E-09 |
| Non-small cell lung cancer | 16/301 | 73/9396 | 8.37989E-10 | 4.78147E-09 | 1.66041E-09 |
| Efferocytosis | 23/301 | 157/9396 | 8.72166E-10 | 4.78869E-09 | 1.66292E-09 |
| Cellular senescence | 23/301 | 157/9396 | 8.72166E-10 | 4.78869E-09 | 1.66292E-09 |
| Influenza A | 24/301 | 173/9396 | 1.15606E-09 | 6.2299E-09 | 2.1634E-09 |
| Platinum drug resistance | 16/301 | 75/9396 | 1.28253E-09 | 6.78576E-09 | 2.35642E-09 |
| Chronic myeloid leukemia | 16/301 | 77/9396 | 1.93513E-09 | 9.92486E-09 | 3.4465E-09 |
| MicroRNAs in cancer | 33/301 | 318/9396 | 1.94404E-09 | 9.92486E-09 | 3.4465E-09 |
| Long-term potentiation | 15/301 | 67/9396 | 2.087E-09 | 1.0471E-08 | 3.63615E-09 |
| cGMP-PKG signaling pathway | 23/301 | 166/9396 | 2.66633E-09 | 1.31509E-08 | 4.56678E-09 |
| Estrogen signaling pathway | 21/301 | 139/9396 | 2.73158E-09 | 1.32481E-08 | 4.60055E-09 |
| Hepatocellular carcinoma | 23/301 | 170/9396 | 4.27052E-09 | 2.03725E-08 | 7.07455E-09 |
| GnRH signaling pathway | 17/301 | 93/9396 | 4.79703E-09 | 2.25151E-08 | 7.81859E-09 |
| Ovarian steroidogenesis | 13/301 | 52/9396 | 5.97257E-09 | 2.75876E-08 | 9.58006E-09 |
| T cell receptor signaling pathway | 19/301 | 122/9396 | 9.51817E-09 | 4.32779E-08 | 1.50287E-08 |
| Phospholipase D signaling pathway | 21/301 | 149/9396 | 9.83241E-09 | 4.4019E-08 | 1.5286E-08 |
| Inflammatory mediator regulation of TRP channels | 17/301 | 99/9396 | 1.28984E-08 | 5.68703E-08 | 1.97488E-08 |
| Legionellosis | 13/301 | 56/9396 | 1.58387E-08 | 6.87919E-08 | 2.38887E-08 |
| Linoleic acid metabolism | 10/301 | 30/9396 | 1.66916E-08 | 7.14303E-08 | 2.48049E-08 |
| Acute myeloid leukemia | 14/301 | 68/9396 | 2.30025E-08 | 9.70104E-08 | 3.36878E-08 |
| Alcoholic liver disease | 20/301 | 144/9396 | 2.87917E-08 | 1.19691E-07 | 4.1564E-08 |
| Adherens junction | 16/301 | 93/9396 | 3.3902E-08 | 1.37021E-07 | 4.75817E-08 |
| Small cell lung cancer | 16/301 | 93/9396 | 3.3902E-08 | 1.37021E-07 | 4.75817E-08 |
| Dopaminergic synapse | 19/301 | 132/9396 | 3.57842E-08 | 1.42647E-07 | 4.95355E-08 |
| TNF signaling pathway | 18/301 | 119/9396 | 3.76467E-08 | 1.48043E-07 | 5.14094E-08 |
| Coronavirus disease - COVID-19 | 26/301 | 238/9396 | 3.91792E-08 | 1.52015E-07 | 5.27888E-08 |
| Chemical carcinogenesis - DNA adducts | 14/301 | 71/9396 | 4.12012E-08 | 1.55708E-07 | 5.40713E-08 |
| Central carbon metabolism in cancer | 14/301 | 71/9396 | 4.12012E-08 | 1.55708E-07 | 5.40713E-08 |
| Human T-cell leukemia virus 1 infection | 25/301 | 224/9396 | 4.7312E-08 | 1.7651E-07 | 6.12948E-08 |
| Chemokine signaling pathway | 23/301 | 193/9396 | 4.92245E-08 | 1.81321E-07 | 6.29654E-08 |
| Steroid hormone biosynthesis | 13/301 | 63/9396 | 7.17004E-08 | 2.6081E-07 | 9.05689E-08 |
| Insulin signaling pathway | 19/301 | 138/9396 | 7.44379E-08 | 2.63684E-07 | 9.1567E-08 |
| Tuberculosis | 22/301 | 182/9396 | 7.52062E-08 | 2.63684E-07 | 9.1567E-08 |
| Progesterone-mediated oocyte maturation | 17/301 | 111/9396 | 7.52089E-08 | 2.63684E-07 | 9.1567E-08 |
| p53 signaling pathway | 14/301 | 75/9396 | 8.54901E-08 | 2.96162E-07 | 1.02845E-07 |
| Osteoclast differentiation | 19/301 | 143/9396 | 1.32766E-07 | 4.54528E-07 | 1.57839E-07 |
| Pertussis | 14/301 | 78/9396 | 1.43145E-07 | 4.84361E-07 | 1.68199E-07 |
| Chagas disease | 16/301 | 103/9396 | 1.49627E-07 | 5.00475E-07 | 1.73795E-07 |
| Type II diabetes mellitus | 11/301 | 47/9396 | 1.8913E-07 | 6.25417E-07 | 2.17182E-07 |
| Gap junction | 15/301 | 92/9396 | 1.9179E-07 | 6.27088E-07 | 2.17762E-07 |
| NF-kappa B signaling pathway | 16/301 | 105/9396 | 1.96879E-07 | 6.36575E-07 | 2.21057E-07 |
| Amphetamine addiction | 13/301 | 69/9396 | 2.227E-07 | 7.12151E-07 | 2.47302E-07 |
| Antifolate resistance | 9/301 | 30/9396 | 2.49432E-07 | 7.88965E-07 | 2.73976E-07 |
| Renal cell carcinoma | 13/301 | 70/9396 | 2.65727E-07 | 8.31153E-07 | 2.88626E-07 |
| Human papillomavirus infection | 30/301 | 333/9396 | 2.68482E-07 | 8.31153E-07 | 2.88626E-07 |
| Regulation of lipolysis in adipocytes | 12/301 | 59/9396 | 2.72647E-07 | 8.3516E-07 | 2.90018E-07 |
| Rap1 signaling pathway | 23/301 | 212/9396 | 2.79042E-07 | 8.45846E-07 | 2.93729E-07 |
| Epithelial cell signaling in Helicobacter pylori infection | 13/301 | 71/9396 | 3.16051E-07 | 9.48153E-07 | 3.29256E-07 |
| Toll-like receptor signaling pathway | 16/301 | 109/9396 | 3.34095E-07 | 9.92058E-07 | 3.44502E-07 |
| Autophagy - animal | 20/301 | 169/9396 | 4.23709E-07 | 1.24545E-06 | 4.32495E-07 |
| Platelet activation | 17/301 | 126/9396 | 4.93337E-07 | 1.43561E-06 | 4.9853E-07 |
| Non-alcoholic fatty liver disease | 19/301 | 157/9396 | 5.85883E-07 | 1.68804E-06 | 5.86188E-07 |
| Diabetic cardiomyopathy | 22/301 | 205/9396 | 6.16649E-07 | 1.75926E-06 | 6.10921E-07 |
| NOD-like receptor signaling pathway | 21/301 | 189/9396 | 6.28396E-07 | 1.77537E-06 | 6.16515E-07 |
| Vascular smooth muscle contraction | 17/301 | 134/9396 | 1.19544E-06 | 3.31309E-06 | 1.1505E-06 |
| Natural killer cell mediated cytotoxicity | 17/301 | 134/9396 | 1.19544E-06 | 3.31309E-06 | 1.1505E-06 |
| Salmonella infection | 24/301 | 251/9396 | 1.55736E-06 | 4.2754E-06 | 1.48467E-06 |
| AMPK signaling pathway | 16/301 | 122/9396 | 1.58263E-06 | 4.30417E-06 | 1.49466E-06 |
| Shigellosis | 24/301 | 253/9396 | 1.79436E-06 | 4.83481E-06 | 1.67894E-06 |
| Th17 cell differentiation | 15/301 | 109/9396 | 1.81831E-06 | 4.85437E-06 | 1.68573E-06 |
| Pathogenic Escherichia coli infection | 21/301 | 203/9396 | 2.03778E-06 | 5.39086E-06 | 1.87203E-06 |
| Epstein-Barr virus infection | 21/301 | 204/9396 | 2.2066E-06 | 5.78486E-06 | 2.00885E-06 |
| Viral carcinogenesis | 21/301 | 205/9396 | 2.38806E-06 | 6.20468E-06 | 2.15464E-06 |
| Cocaine addiction | 10/301 | 49/9396 | 2.6588E-06 | 6.84701E-06 | 2.37769E-06 |
| Colorectal cancer | 13/301 | 87/9396 | 3.51213E-06 | 8.96517E-06 | 3.11324E-06 |
| Melanogenesis | 14/301 | 101/9396 | 3.69384E-06 | 9.34701E-06 | 3.24584E-06 |
| Metabolism of xenobiotics by cytochrome P450 | 12/301 | 79/9396 | 7.02789E-06 | 1.76303E-05 | 6.1223E-06 |
| Focal adhesion | 20/301 | 203/9396 | 7.56056E-06 | 1.88045E-05 | 6.53004E-06 |
| Oxytocin signaling pathway | 17/301 | 155/9396 | 8.97219E-06 | 2.21263E-05 | 7.68359E-06 |
| Renin secretion | 11/301 | 69/9396 | 1.05651E-05 | 2.58356E-05 | 8.97166E-06 |
| Cell cycle | 17/301 | 158/9396 | 1.16009E-05 | 2.81322E-05 | 9.76919E-06 |
| Adipocytokine signaling pathway | 11/301 | 70/9396 | 1.21825E-05 | 2.92984E-05 | 1.01742E-05 |
| Choline metabolism in cancer | 13/301 | 99/9396 | 1.50398E-05 | 3.58737E-05 | 1.24575E-05 |
| Endometrial cancer | 10/301 | 59/9396 | 1.53055E-05 | 3.62106E-05 | 1.25745E-05 |
| Neutrophil extracellular trap formation | 19/301 | 196/9396 | 1.61601E-05 | 3.79242E-05 | 1.31696E-05 |
| Drug metabolism - cytochrome P450 | 11/301 | 73/9396 | 1.83927E-05 | 4.24784E-05 | 1.4751E-05 |
| Melanoma | 11/301 | 73/9396 | 1.83927E-05 | 4.24784E-05 | 1.4751E-05 |
| Aldosterone-regulated sodium reabsorption | 8/301 | 38/9396 | 2.1371E-05 | 4.86899E-05 | 1.6908E-05 |
| Folate biosynthesis | 7/301 | 28/9396 | 2.14169E-05 | 4.86899E-05 | 1.6908E-05 |
| Longevity regulating pathway | 12/301 | 90/9396 | 2.74364E-05 | 6.18914E-05 | 2.14924E-05 |
| B cell receptor signaling pathway | 12/301 | 91/9396 | 3.0713E-05 | 6.87498E-05 | 2.3874E-05 |
| Oocyte meiosis | 15/301 | 139/9396 | 3.67722E-05 | 8.11103E-05 | 2.81664E-05 |
| GnRH secretion | 10/301 | 65/9396 | 3.67923E-05 | 8.11103E-05 | 2.81664E-05 |
| Wnt signaling pathway | 17/301 | 174/9396 | 4.0963E-05 | 8.96257E-05 | 3.11234E-05 |
| Folate transport and metabolism | 7/301 | 31/9396 | 4.3791E-05 | 9.50983E-05 | 3.30238E-05 |
| mTOR signaling pathway | 16/301 | 158/9396 | 4.46031E-05 | 9.61444E-05 | 3.33871E-05 |
| Fc gamma R-mediated phagocytosis | 12/301 | 99/9396 | 7.15933E-05 | 0.000153189 | 5.31963E-05 |
| Herpes simplex virus 1 infection | 17/301 | 182/9396 | 7.23918E-05 | 0.000153767 | 5.33969E-05 |
| Breast cancer | 15/301 | 148/9396 | 7.63775E-05 | 0.000161057 | 5.59286E-05 |
| Axon guidance | 17/301 | 184/9396 | 8.29886E-05 | 0.000173739 | 6.03325E-05 |
| Amoebiasis | 12/301 | 103/9396 | 0.000105651 | 0.000219603 | 7.62593E-05 |
| Adrenergic signaling in cardiomyocytes | 15/301 | 154/9396 | 0.000120123 | 0.000247912 | 8.60901E-05 |
| Cushing syndrome | 15/301 | 155/9396 | 0.000129218 | 0.000264805 | 9.19563E-05 |
| Bile secretion | 11/301 | 90/9396 | 0.00013258 | 0.000269795 | 9.36891E-05 |
| Gastric acid secretion | 10/301 | 76/9396 | 0.000143738 | 0.000290471 | 0.000100869 |
| Apelin signaling pathway | 14/301 | 140/9396 | 0.000152603 | 0.000304533 | 0.000105752 |
| Glucagon signaling pathway | 12/301 | 107/9396 | 0.00015279 | 0.000304533 | 0.000105752 |
| Parkinson disease | 21/301 | 271/9396 | 0.000160855 | 0.000318427 | 0.000110577 |
| Leishmaniasis | 10/301 | 79/9396 | 0.000199328 | 0.000391923 | 0.000136099 |
| Prion disease | 21/301 | 278/9396 | 0.000228944 | 0.000447132 | 0.000155271 |
| Transcriptional misregulation in cancer | 17/301 | 201/9396 | 0.00024373 | 0.000472836 | 0.000164197 |
| Parathyroid hormone synthesis, secretion and action | 12/301 | 115/9396 | 0.00030259 | 0.000583137 | 0.0002025 |
| Gastric cancer | 14/301 | 150/9396 | 0.000315125 | 0.000603299 | 0.000209502 |
| RIG-I-like receptor signaling pathway | 9/301 | 72/9396 | 0.000455414 | 0.000866179 | 0.000300789 |
| Necroptosis | 14/301 | 159/9396 | 0.000570905 | 0.001078788 | 0.00037462 |
| Long-term depression | 8/301 | 60/9396 | 0.000606729 | 0.001139085 | 0.000395559 |
| Longevity regulating pathway - multiple species | 8/301 | 62/9396 | 0.000758604 | 0.001415088 | 0.000491403 |
| Tyrosine metabolism | 6/301 | 36/9396 | 0.000889687 | 0.001649037 | 0.000572645 |
| Thyroid cancer | 6/301 | 37/9396 | 0.001033746 | 0.001903925 | 0.000661157 |
| Salivary secretion | 10/301 | 97/9396 | 0.001048181 | 0.001918368 | 0.000666172 |
| Aldosterone synthesis and secretion | 10/301 | 98/9396 | 0.00113496 | 0.002064208 | 0.000716817 |
| Leukocyte transendothelial migration | 11/301 | 116/9396 | 0.001199251 | 0.002166099 | 0.000752199 |
| Alcoholism | 15/301 | 191/9396 | 0.00120587 | 0.002166099 | 0.000752199 |
| Mitophagy - animal | 10/301 | 105/9396 | 0.001920887 | 0.003429313 | 0.001190863 |
| Signaling pathways regulating pluripotency of stem cells | 12/301 | 144/9396 | 0.002230654 | 0.003958051 | 0.001374473 |
| Th1 and Th2 cell differentiation | 9/301 | 93/9396 | 0.002866525 | 0.005055508 | 0.001755575 |
| Apoptosis - multiple species | 5/301 | 32/9396 | 0.003227942 | 0.00565862 | 0.001965012 |
| Circadian entrainment | 9/301 | 97/9396 | 0.003811745 | 0.006642022 | 0.002306508 |
| Inflammatory bowel disease | 7/301 | 66/9396 | 0.004986321 | 0.00863702 | 0.002999291 |
| Renin-angiotensin system | 4/301 | 23/9396 | 0.005647015 | 0.009723558 | 0.003376602 |
| African trypanosomiasis | 5/301 | 37/9396 | 0.006134357 | 0.010465014 | 0.00363408 |
| Insulin secretion | 8/301 | 86/9396 | 0.006149544 | 0.010465014 | 0.00363408 |
| GABAergic synapse | 8/301 | 89/9396 | 0.007540131 | 0.012756849 | 0.004429942 |
| Regulation of actin cytoskeleton | 15/301 | 232/9396 | 0.007640338 | 0.012851667 | 0.004462869 |
| JAK-STAT signaling pathway | 12/301 | 168/9396 | 0.007712998 | 0.012899324 | 0.004479418 |
| Retrograde endocannabinoid signaling | 11/301 | 149/9396 | 0.008316341 | 0.013828886 | 0.004802218 |
| Morphine addiction | 8/301 | 91/9396 | 0.008590702 | 0.014203944 | 0.00493246 |
| Hedgehog signaling pathway | 6/301 | 56/9396 | 0.008695329 | 0.01429571 | 0.004964327 |
| Tryptophan metabolism | 5/301 | 42/9396 | 0.010516109 | 0.017192067 | 0.005970115 |
| PPAR signaling pathway | 7/301 | 76/9396 | 0.010685078 | 0.017370714 | 0.006032152 |
| Thiamine metabolism | 3/301 | 15/9396 | 0.011124331 | 0.017984335 | 0.006245238 |
| Glutamatergic synapse | 9/301 | 116/9396 | 0.012028125 | 0.019338035 | 0.006715324 |
| Base excision repair | 5/301 | 44/9396 | 0.01274916 | 0.020384646 | 0.00707877 |
| Phenylalanine metabolism | 3/301 | 16/9396 | 0.013371928 | 0.021263558 | 0.007383981 |
| ABC transporters | 5/301 | 45/9396 | 0.013977389 | 0.022105544 | 0.007676369 |
| Drug metabolism - other enzymes | 7/301 | 81/9396 | 0.014857094 | 0.023369807 | 0.008115397 |
| Cytosolic DNA-sensing pathway | 7/301 | 83/9396 | 0.016812938 | 0.026304113 | 0.009134364 |
| Spinocerebellar ataxia | 10/301 | 144/9396 | 0.017239411 | 0.026827105 | 0.009315978 |
| Pancreatic secretion | 8/301 | 106/9396 | 0.020264261 | 0.03136649 | 0.010892324 |
| Retinol metabolism | 6/301 | 68/9396 | 0.021351744 | 0.032874908 | 0.011416137 |
| Fructose and mannose metabolism | 4/301 | 34/9396 | 0.022474938 | 0.034422141 | 0.011953429 |
| Pentose and glucuronate interconversions | 4/301 | 36/9396 | 0.027167587 | 0.041391455 | 0.014373593 |
| Proximal tubule bicarbonate reclamation | 3/301 | 23/9396 | 0.035885952 | 0.054389646 | 0.018887343 |
| Glutathione metabolism | 5/301 | 59/9396 | 0.040008305 | 0.060323403 | 0.020947899 |
| Protein processing in endoplasmic reticulum | 10/301 | 171/9396 | 0.048119555 | 0.072179333 | 0.025064988 |
| alpha-Linolenic acid metabolism | 3/301 | 26/9396 | 0.049135208 | 0.073324849 | 0.02546278 |

Supplementary Table S5. Detailed KEGG pathway enrichment results for individual metabolites.

| **Q-OS\|THP-Ac** | **Q-OS\|PCA** | **Q-OS\|BZF** | **Q-OS\|Q-dimer** |
| --- | --- | --- | --- |
| Lipid and atherosclerosis | Lipid and atherosclerosis | Nitrogen metabolism | Nitrogen metabolism |
| Chemical carcinogenesis - reactive oxygen species | Nitrogen metabolism | Serotonergic synapse | Lipid and atherosclerosis |
| HIF-1 signaling pathway | HIF-1 signaling pathway | HIF-1 signaling pathway | Chemical carcinogenesis - reactive oxygen species |
| Nitrogen metabolism | Chemical carcinogenesis - receptor activation | Chemical carcinogenesis - reactive oxygen species | Chemical carcinogenesis - receptor activation |
| AGE-RAGE signaling pathway in diabetic complications | AGE-RAGE signaling pathway in diabetic complications | Chemical carcinogenesis - receptor activation | Arachidonic acid metabolism |
| Chemical carcinogenesis - receptor activation | Serotonergic synapse | AGE-RAGE signaling pathway in diabetic complications | C-type lectin receptor signaling pathway |
| Hepatitis B | Chemical carcinogenesis - reactive oxygen species | MAPK signaling pathway | Prostate cancer |
| Serotonergic synapse | Prostate cancer | Arachidonic acid metabolism | HIF-1 signaling pathway |
| PD-L1 expression and PD-1 checkpoint pathway in cancer | Fluid shear stress and atherosclerosis | Ovarian steroidogenesis | Serotonergic synapse |
| Sphingolipid signaling pathway | Calcium signaling pathway | Lipid and atherosclerosis | Fluid shear stress and atherosclerosis |
| Proteoglycans in cancer | PD-L1 expression and PD-1 checkpoint pathway in cancer | Neurotrophin signaling pathway | Sphingolipid signaling pathway |
| Neurotrophin signaling pathway | Sphingolipid signaling pathway | Sphingolipid signaling pathway | Chemical carcinogenesis - DNA adducts |
| Apoptosis | Arachidonic acid metabolism | Prostate cancer | Linoleic acid metabolism |
| Thyroid hormone signaling pathway | Ovarian steroidogenesis | cAMP signaling pathway | Antifolate resistance |
| Prostate cancer | cAMP signaling pathway | Proteoglycans in cancer | PI3K-Akt signaling pathway |
| Kaposi sarcoma-associated herpesvirus infection | Thyroid hormone signaling pathway | PI3K-Akt signaling pathway | Endocrine resistance |
| Toxoplasmosis | Insulin resistance | Linoleic acid metabolism | AGE-RAGE signaling pathway in diabetic complications |
| Human immunodeficiency virus 1 infection | IL-17 signaling pathway | Chemical carcinogenesis - DNA adducts | Kaposi sarcoma-associated herpesvirus infection |
| Growth hormone synthesis, secretion and action | Hepatitis B | Glioma | PD-L1 expression and PD-1 checkpoint pathway in cancer |
| Human cytomegalovirus infection | Cholinergic synapse | Thyroid hormone signaling pathway | MAPK signaling pathway |
| MicroRNAs in cancer | Proteoglycans in cancer | Long-term potentiation | Drug metabolism - cytochrome P450 |
| Fc epsilon RI signaling pathway | Linoleic acid metabolism | ErbB signaling pathway | Small cell lung cancer |
| Prolactin signaling pathway | Steroid hormone biosynthesis | C-type lectin receptor signaling pathway | Yersinia infection |
| Influenza A | Growth hormone synthesis, secretion and action | Hepatocellular carcinoma | Human cytomegalovirus infection |
| Non-small cell lung cancer | Bladder cancer | Adherens junction | Neuroactive ligand signaling |
| VEGF signaling pathway | Efferocytosis | Cholinergic synapse | Metabolism of xenobiotics by cytochrome P450 |
| Estrogen signaling pathway | Pathways of neurodegeneration - multiple diseases | Chronic myeloid leukemia | Proteoglycans in cancer |
| C-type lectin receptor signaling pathway | Fc epsilon RI signaling pathway | Estrogen signaling pathway | Steroid hormone biosynthesis |
| Alzheimer disease | Endocrine resistance | Ras signaling pathway | Folate transport and metabolism |
| Fluid shear stress and atherosclerosis | Chemical carcinogenesis - DNA adducts | Hepatitis B | Cellular senescence |
| cAMP signaling pathway | Type II diabetes mellitus | Endocrine resistance | Human immunodeficiency virus 1 infection |
| Pancreatic cancer | Non-small cell lung cancer | Pathways of neurodegeneration - multiple diseases | Neuroactive ligand-receptor interaction |
| Steroid hormone biosynthesis | Neurotrophin signaling pathway | Fluid shear stress and atherosclerosis | Cocaine addiction |
| Arachidonic acid metabolism | Alzheimer disease | EGFR tyrosine kinase inhibitor resistance | Bile secretion |
| Insulin resistance | Estrogen signaling pathway | Steroid hormone biosynthesis | Estrogen signaling pathway |
| MAPK signaling pathway | Hormone signaling | Alzheimer disease | Measles |
| EGFR tyrosine kinase inhibitor resistance | Neuroactive ligand signaling | Cocaine addiction | cGMP-PKG signaling pathway |
| Bladder cancer | Adherens junction | Phospholipase D signaling pathway | Prolactin signaling pathway |
| Long-term potentiation | Toxoplasmosis | Amphetamine addiction | Central carbon metabolism in cancer |
| Cholinergic synapse | Relaxin signaling pathway | Insulin resistance | Ovarian steroidogenesis |
| Endocrine resistance | Phospholipase D signaling pathway | MicroRNAs in cancer | cAMP signaling pathway |
| Renal cell carcinoma | Autophagy - animal | Dopaminergic synapse | Neurotrophin signaling pathway |
| TNF signaling pathway | FoxO signaling pathway | PD-L1 expression and PD-1 checkpoint pathway in cancer | Pancreatic cancer |
| Efferocytosis | MAPK signaling pathway | Prolactin signaling pathway | EGFR tyrosine kinase inhibitor resistance |
| Central carbon metabolism in cancer | Human immunodeficiency virus 1 infection | Progesterone-mediated oocyte maturation | Bladder cancer |
| Hepatitis C | Prolactin signaling pathway | Cellular senescence | VEGF signaling pathway |
| Pathways of neurodegeneration - multiple diseases | Central carbon metabolism in cancer | Toxoplasmosis | Relaxin signaling pathway |
| T cell receptor signaling pathway | Apoptosis | Gap junction | Efferocytosis |
| Osteoclast differentiation | Ras signaling pathway | Drug metabolism - cytochrome P450 | Hepatitis C |
| Platinum drug resistance | Regulation of lipolysis in adipocytes | Bladder cancer | Hormone signaling |
| Toll-like receptor signaling pathway | C-type lectin receptor signaling pathway | IL-17 signaling pathway | Insulin resistance |
| Glioma | VEGF signaling pathway | Pancreatic cancer | MicroRNAs in cancer |
| Chronic myeloid leukemia | Glioma | VEGF signaling pathway | Hepatitis B |
| Relaxin signaling pathway | MicroRNAs in cancer | Kaposi sarcoma-associated herpesvirus infection | Calcium signaling pathway |
| Ras signaling pathway | Pancreatic cancer | Leishmaniasis | Progesterone-mediated oocyte maturation |
| IL-17 signaling pathway | GnRH signaling pathway | T cell receptor signaling pathway | Apoptosis |
| Ovarian steroidogenesis | Aldosterone-regulated sodium reabsorption | Growth hormone synthesis, secretion and action | Toxoplasmosis |
| Yersinia infection | Hepatocellular carcinoma | Calcium signaling pathway | Acute myeloid leukemia |
| Acute myeloid leukemia | EGFR tyrosine kinase inhibitor resistance | Melanogenesis | Human papillomavirus infection |
| Measles | Coronavirus disease - COVID-19 | Human cytomegalovirus infection | Adherens junction |
| Linoleic acid metabolism | Long-term potentiation | Antifolate resistance | Renal cell carcinoma |
| Antifolate resistance | Kaposi sarcoma-associated herpesvirus infection | Diabetic cardiomyopathy | Pathways of neurodegeneration - multiple diseases |
| Chagas disease | Acute myeloid leukemia | Type II diabetes mellitus | Epithelial cell signaling in Helicobacter pylori infection |
| Legionellosis | Amphetamine addiction | Relaxin signaling pathway | Ras signaling pathway |
| Human papillomavirus infection | Measles | Oxytocin signaling pathway | IL-17 signaling pathway |
| FoxO signaling pathway | Human T-cell leukemia virus 1 infection | Tuberculosis | Tuberculosis |
| Natural killer cell mediated cytotoxicity | Human cytomegalovirus infection | FoxO signaling pathway | Herpes simplex virus 1 infection |
| Human T-cell leukemia virus 1 infection | Diabetic cardiomyopathy | Hepatitis C | p53 signaling pathway |
| Cellular senescence | Osteoclast differentiation | Human immunodeficiency virus 1 infection | Glioma |
| Non-alcoholic fatty liver disease | Drug metabolism - cytochrome P450 | Fc epsilon RI signaling pathway | Chronic myeloid leukemia |
| Insulin signaling pathway | Longevity regulating pathway | Bile secretion | Alzheimer disease |
| GnRH secretion | cGMP-PKG signaling pathway | Apoptosis | Dopaminergic synapse |
| Coronavirus disease - COVID-19 | Th17 cell differentiation | Central carbon metabolism in cancer | Transcriptional misregulation in cancer |
| ErbB signaling pathway | Platinum drug resistance | Oocyte meiosis | ErbB signaling pathway |
| Chemokine signaling pathway | Chemokine signaling pathway | GnRH signaling pathway | Tryptophan metabolism |
| Hepatocellular carcinoma | PI3K-Akt signaling pathway | Chemokine signaling pathway | Epstein-Barr virus infection |
| Epithelial cell signaling in Helicobacter pylori infection | Vascular smooth muscle contraction | Non-small cell lung cancer | ABC transporters |
| Gap junction | Cocaine addiction | Human T-cell leukemia virus 1 infection | Phospholipase D signaling pathway |
| Salmonella infection | Neuroactive ligand-receptor interaction | Legionellosis | Retinol metabolism |
| GnRH signaling pathway | Inflammatory mediator regulation of TRP channels | Influenza A | AMPK signaling pathway |
| Endometrial cancer | Choline metabolism in cancer | Inflammatory mediator regulation of TRP channels | T cell receptor signaling pathway |
| Vascular smooth muscle contraction | Insulin signaling pathway | Metabolism of xenobiotics by cytochrome P450 | Thyroid hormone signaling pathway |
| Pathogenic Escherichia coli infection | Yersinia infection | Axon guidance | Fc epsilon RI signaling pathway |
| Pertussis | Hepatitis C | Efferocytosis | Adipocytokine signaling pathway |
| Type II diabetes mellitus | TNF signaling pathway | Cell cycle | Folate biosynthesis |
| Choline metabolism in cancer | T cell receptor signaling pathway | Folate biosynthesis | Platelet activation |
| Cocaine addiction | ErbB signaling pathway | Vascular smooth muscle contraction | NOD-like receptor signaling pathway |
| Alcoholic liver disease | NF-kappa B signaling pathway | Natural killer cell mediated cytotoxicity | Melanoma |
| Autophagy - animal | Adipocytokine signaling pathway | Th17 cell differentiation | Non-small cell lung cancer |
| NF-kappa B signaling pathway | Renal cell carcinoma | Salmonella infection | Chemokine signaling pathway |
| Platelet activation | Platelet activation | Yersinia infection | FoxO signaling pathway |
| Neutrophil extracellular trap formation | Legionellosis | Measles | NF-kappa B signaling pathway |
| Phospholipase D signaling pathway | Gap junction | Longevity regulating pathway | Pertussis |
| Colorectal cancer | Antifolate resistance | Acute myeloid leukemia | Leishmaniasis |
| Shigellosis | Wnt signaling pathway | Folate transport and metabolism | Autophagy - animal |
| Th17 cell differentiation | Oxytocin signaling pathway | Neuroactive ligand signaling | Phenylalanine metabolism |
| Calcium signaling pathway | Natural killer cell mediated cytotoxicity | Renal cell carcinoma | Oocyte meiosis |
| Chemical carcinogenesis - DNA adducts | Chronic myeloid leukemia | Pathogenic Escherichia coli infection | Toll-like receptor signaling pathway |
| Progesterone-mediated oocyte maturation | Metabolism of xenobiotics by cytochrome P450 | Human papillomavirus infection | Legionellosis |
| B cell receptor signaling pathway | Human papillomavirus infection | Viral carcinogenesis | Influenza A |
| Tuberculosis | GnRH secretion | Melanoma | Osteoclast differentiation |
| Rap1 signaling pathway | AMPK signaling pathway | Breast cancer | Pentose and glucuronate interconversions |
| Regulation of lipolysis in adipocytes | Tyrosine metabolism | Platinum drug resistance | Rap1 signaling pathway |
| NOD-like receptor signaling pathway | Renin secretion | p53 signaling pathway | Cholinergic synapse |
| Leishmaniasis | Influenza A | Tyrosine metabolism | Salmonella infection |
| Inflammatory mediator regulation of TRP channels | Toll-like receptor signaling pathway | Platelet activation | Breast cancer |
| Folate transport and metabolism | Bile secretion | Cushing syndrome | Shigellosis |
| Amoebiasis | Dopaminergic synapse | Pertussis | Gastric cancer |
| Epstein-Barr virus infection | Transcriptional misregulation in cancer | Chagas disease | Longevity regulating pathway |
| Amphetamine addiction | Progesterone-mediated oocyte maturation | Amoebiasis | Gap junction |
| Bile secretion | Cellular senescence | mTOR signaling pathway | Human T-cell leukemia virus 1 infection |
| Adipocytokine signaling pathway | Parathyroid hormone synthesis, secretion and action | Aldosterone-regulated sodium reabsorption | Cell cycle |
| Thyroid cancer | Leukocyte transendothelial migration | NOD-like receptor signaling pathway | Proximal tubule bicarbonate reclamation |
| PI3K-Akt signaling pathway | Gastric acid secretion | Regulation of lipolysis in adipocytes | Coronavirus disease - COVID-19 |
| Adherens junction | Endometrial cancer | Endometrial cancer | Focal adhesion |
| Small cell lung cancer | Long-term depression | Long-term depression | Pathogenic Escherichia coli infection |
| Drug metabolism - cytochrome P450 | NOD-like receptor signaling pathway | Toll-like receptor signaling pathway | Chagas disease |
| Melanoma | Alcoholic liver disease | cGMP-PKG signaling pathway | Viral carcinogenesis |
| Aldosterone-regulated sodium reabsorption | Longevity regulating pathway - multiple species | Tryptophan metabolism | Hepatocellular carcinoma |
| p53 signaling pathway | Melanogenesis | Apelin signaling pathway | Platinum drug resistance |
| Fc gamma R-mediated phagocytosis | Chagas disease | Colorectal cancer | Mitophagy - animal |
| Neuroactive ligand signaling | Folate transport and metabolism | GnRH secretion | Arginine and proline metabolism |
| Metabolism of xenobiotics by cytochrome P450 | Mitophagy - animal | Osteoclast differentiation | Alcoholic liver disease |
| Melanogenesis | Shigellosis | Inflammatory bowel disease | Axon guidance |
| Viral carcinogenesis | Viral carcinogenesis | Parkinson disease | TNF signaling pathway |
| Diabetic cardiomyopathy | Non-alcoholic fatty liver disease | Coronavirus disease - COVID-19 | Colorectal cancer |
| Oxytocin signaling pathway | mTOR signaling pathway | B cell receptor signaling pathway | Regulation of actin cytoskeleton |
| Herpes simplex virus 1 infection | Axon guidance | Morphine addiction | Regulation of lipolysis in adipocytes |
| Dopaminergic synapse | Epithelial cell signaling in Helicobacter pylori infection | Retinol metabolism | Endometrial cancer |
| mTOR signaling pathway | Melanoma | Small cell lung cancer | B cell receptor signaling pathway |
| Hormone signaling | Thyroid cancer | Gastric cancer | Fructose and mannose metabolism |
| Longevity regulating pathway | PPAR signaling pathway | Rap1 signaling pathway | Tyrosine metabolism |
| cGMP-PKG signaling pathway | Fc gamma R-mediated phagocytosis | Adipocytokine signaling pathway | JAK-STAT signaling pathway |
| Th1 and Th2 cell differentiation | Neutrophil extracellular trap formation | Epithelial cell signaling in Helicobacter pylori infection | Thyroid cancer |
| RIG-I-like receptor signaling pathway | Pertussis | Aldosterone synthesis and secretion | Inflammatory bowel disease |
| Focal adhesion | Prion disease | Hormone signaling | Thiamine metabolism |
| Breast cancer | Leishmaniasis | Fc gamma R-mediated phagocytosis | Natural killer cell mediated cytotoxicity |
| Gastric acid secretion | Breast cancer | Thiamine metabolism | Renin secretion |
| Gastric cancer | Drug metabolism - other enzymes | Alcoholism | Amphetamine addiction |
| Adrenergic signaling in cardiomyocytes | Amoebiasis | Gastric acid secretion | Insulin signaling pathway |
| Axon guidance | Focal adhesion | Phenylalanine metabolism | Amoebiasis |
| Cell cycle | Base excision repair | Neutrophil extracellular trap formation | Apelin signaling pathway |
| Prion disease | Glucagon signaling pathway | NF-kappa B signaling pathway | RIG-I-like receptor signaling pathway |
| Long-term depression | Tuberculosis | Hedgehog signaling pathway | Th17 cell differentiation |
| Tryptophan metabolism | Folate biosynthesis | Insulin signaling pathway | Base excision repair |
| JAK-STAT signaling pathway | Insulin secretion | Focal adhesion | Pyruvate metabolism |
| Inflammatory bowel disease | Rap1 signaling pathway | Pentose and glucuronate interconversions | Type II diabetes mellitus |
| Parathyroid hormone synthesis, secretion and action | Colorectal cancer | Wnt signaling pathway | Drug metabolism - other enzymes |
| Glutamatergic synapse | GABAergic synapse | Thyroid cancer | Neutrophil extracellular trap formation |
| Signaling pathways regulating pluripotency of stem cells | Retinol metabolism | Prion disease |  |
| Folate biosynthesis | B cell receptor signaling pathway | Alcoholic liver disease |  |
| Renin secretion | Apelin signaling pathway | GABAergic synapse |  |
| AMPK signaling pathway | Phenylalanine metabolism | TNF signaling pathway |  |
| Circadian entrainment | Glutamatergic synapse | Proximal tubule bicarbonate reclamation |  |
| Phenylalanine metabolism | Th1 and Th2 cell differentiation | Renin secretion |  |
| Necroptosis | Small cell lung cancer | Circadian entrainment |  |
| Pancreatic secretion | JAK-STAT signaling pathway | ABC transporters |  |
| Hedgehog signaling pathway | Salmonella infection | Choline metabolism in cancer |  |
| Glucagon signaling pathway | RIG-I-like receptor signaling pathway | Autophagy - animal |  |
| Pentose and glucuronate interconversions | Salivary secretion | Arginine and proline metabolism |  |
| Tyrosine metabolism | Fructose and mannose metabolism | Endocrine and other factor-regulated calcium reabsorption |  |
| African trypanosomiasis | Aldosterone synthesis and secretion | Signaling pathways regulating pluripotency of stem cells |  |
| Oocyte meiosis | p53 signaling pathway | Shigellosis |  |
| Apelin signaling pathway | Retrograde endocannabinoid signaling | Herpes simplex virus 1 infection |  |
| Parkinson disease | Adrenergic signaling in cardiomyocytes | Parathyroid hormone synthesis, secretion and action |  |
| Insulin secretion | African trypanosomiasis | Retrograde endocannabinoid signaling |  |
| Spinocerebellar ataxia | Cushing syndrome | Glutamatergic synapse |  |
| Longevity regulating pathway - multiple species | Parkinson disease | Insulin secretion |  |
| Leukocyte transendothelial migration | Glutathione metabolism | Adrenergic signaling in cardiomyocytes |  |
| GABAergic synapse | Pancreatic secretion | AMPK signaling pathway |  |
| Retrograde endocannabinoid signaling | Necroptosis | Fructose and mannose metabolism |  |
| Proximal tubule bicarbonate reclamation | Alcoholism | Necroptosis |  |
| Retinol metabolism | Renin-angiotensin system | Longevity regulating pathway - multiple species |  |
| Base excision repair | Proximal tubule bicarbonate reclamation | Th1 and Th2 cell differentiation |  |
| Cushing syndrome | Tryptophan metabolism | African trypanosomiasis |  |
| Salivary secretion | Inflammatory bowel disease | Salivary secretion |  |
| Aldosterone synthesis and secretion | Morphine addiction | Epstein-Barr virus infection |  |
| Alcoholism | ABC transporters | Neuroactive ligand-receptor interaction |  |
| Neuroactive ligand-receptor interaction | Pathogenic Escherichia coli infection | Pancreatic secretion |  |
| Thyroid hormone synthesis | Epstein-Barr virus infection | Thyroid hormone synthesis |  |
| Vibrio cholerae infection | Gastric cancer | TGF-beta signaling pathway |  |
| Protein processing in endoplasmic reticulum | Circadian entrainment |  |  |
| Endocrine and other factor-regulated calcium reabsorption | Herpes simplex virus 1 infection |  |  |
| Drug metabolism - other enzymes | Thyroid hormone synthesis |  |  |
| Cytosolic DNA-sensing pathway | Vibrio cholerae infection |  |  |
| Fructose and mannose metabolism | Cell cycle |  |  |
| Rheumatoid arthritis | Endocrine and other factor-regulated calcium reabsorption |  |  |
| Transcriptional misregulation in cancer | Antigen processing and presentation |  |  |
| Phosphatidylinositol signaling system | Hedgehog signaling pathway |  |  |
|  | Regulation of actin cytoskeleton |  |  |
|  | Thiamine metabolism |  |  |
|  | Protein processing in endoplasmic reticulum |  |  |
